# *In silico* identification of *Leishmania* GP63 protein epitopes to generate a new vaccine antigen against leishmaniasis

**DOI:** 10.1371/journal.pntd.0013137

**Published:** 2025-06-05

**Authors:** Mervenur Güvendi, Hüseyin Can, İrem Yavuz, Ahmet Özbilgin, Aysu Değirmenci Döşkaya, Muhammet Karakavuk, Cemal Ün, Adnan Yüksel Gürüz, İsmail Cem Yılmaz, Mayda Gürsel, İhsan Gürsel, Mert Döşkaya

**Affiliations:** 1 Department of Biology Molecular Biology Section, Faculty of Science, Ege University, İzmir, Türkiye; 2 Vaccine Development, Application and Research Center, Ege University, İzmir, Türkiye; 3 Department of Vaccine Studies, Institute of Health Sciences, Ege University, İzmir, Türkiye; 4 Department of Parasitology, Faculty of Medicine, Celal Bayar University, Manisa, Türkiye; 5 Department of Parasitology, Faculty of Medicine, Ege University, İzmir, Türkiye; 6 Ödemiş Vocational School, Ege University, İzmir, Türkiye; 7 İzmir Biomedicine and Genome Center, İzmir, Türkiye; Centro de Pesquisa Gonçalo Moniz-FIOCRUZ/BA, BRAZIL

## Abstract

**Background:**

The surface of *Leishmania* spp. presents glycoprotein 63 (GP63), a metalloprotease that acts as one of the parasite’s major antigens. A vaccine against leishmaniasis has not yet been developed and stationary phase promastigotes have utmost importance in transmitting *Leishmania* spp. from phlebotomine sand fly to humans or reservoirs. Therefore, this study aimed to analyze GP63 protein in three different *Leishmania* spp. to determine new vaccine candidate antigen against leishmaniasis using sequencing data of locally detected *Leishmania* strains and *in silico* approaches.

**Methodology/Principal findings:**

The GP63 protein sequences of the stationary phase/amastigote form of *L. infantum*, *L. major*, and *L. tropica* were identified and then the gene encoding GP63 protein in *Leishmania* positive samples (n:59) was amplified and sequenced for variation analysis. According to the results, 4, 6, 19 GP63 variants were found within *L. infantum*, *L. major*, and *L. tropica* isolates, respectively. The most prevalent variants within each species were selected for further analysis using *in silico* approaches. Accordingly, all selected GP63 proteins were antigenic and the amount of B and T cell epitopes were 23 for *L. infantum*, 10 for *L. major*, and 9 for *L. tropica*. The analysis of each epitope showed that all of them were non-toxic, non-allergen, and soluble but had different antigenicity values. Among these epitopes, EMEDQGSAGSAGS associated with *L. major*, STHDSGSTTC and AEDILTDEKRDILRK epitopes associated with *L. infantum* had the highest antigenicity values for B cell, MHC-I, and MHC-II epitopes, respectively. Moreover, conserved epitopes were detected among two or three *Leishmania* species.

**Conclusions/Significance:**

This study detected many epitopes that could be used in vaccine studies and the development of serological diagnostic assays.

## Author summary

Leishmaniasis is a neglected parasitic disease that causes significant health problems in humans as well as canine reservoirs. A safe and protective vaccine against leishmaniasis has not yet been developed. *Leishmania* spp. express glycoprotein 63 (GP63) which is a metalloprotease that acts as one of the parasite’s major surface antigens. In this study, we aimed to analyze the multi-copy GP63 gene in three different *Leishmania* species and determine a novel vaccine candidate antigen against leishmaniasis using sequencing data of locally detected *Leishmania* strains isolated from humans and *in silico* approaches. According to the results, the GP63 proteins selected for each *Leishmania* species were antigenic and contained many B and T cell epitopes. Each epitope selected was non-toxic, non-allergenic and soluble, with different antigenicity values. Among them, the epitopes EMEDQGSAGSAGSAGS, STHDSGSTTC and AEDILTDEKRDILRK were found to be more suitable for the development of vaccines and serologic tests. Furthermore, conserved epitopes identified between two or three *Leishmania* species can be valuable for vaccine development purposes.

## Introduction

Leishmaniasis is a vector-borne infectious disease caused by obligate intracellular protozoan parasites of the genus *Leishmania* [1]. There are 22 different species within the genus *Leishmania* [2]. Each *Leishmania* species is characterized by host factors, specific geographical preferences, and the symptoms occurred during the disease they cause. Leishmaniasis presents in three main clinical forms: cutaneous leishmaniasis (CL), visceral leishmaniasis (VL), and mucocutaneous leishmaniasis (MCL). The other two rare types are disseminated cutaneous leishmaniasis (DCL) and post-kala-azar dermal leishmaniasis (PKDL) [3]. VL, also known as kala-azar, is the most lethal form of leishmaniasis and fatal in over 95% of untreated cases. VL is caused by *L. infantum*, *L. chagasi*, and *L. donovani*. CL is the most common form which causes ulcerative skin lesions on exposed parts of the body. CL is typically caused by *L. tropica*, *L. major*, *L. aethiopica*, *L. amazonensis*, *L. mexicana*, *L. braziliensis*, *L. guyanensis*, and *L. panamensis* [4]. All *Leishmania* species, including those that infect mammals, are transmitted by phlebotomine sand flies.

**Fig 1:**
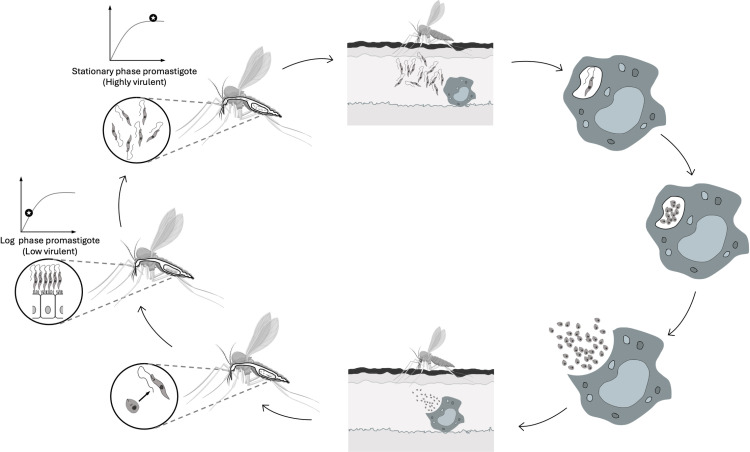
Life cycle of *Leishmania* spp. demonstrating specifically the log and stationary phase of the promastigotes in the gut of the sandfly. Accordingly, the infected sand fly inoculates the stationary phase promastigotes into the skin and the promastigotes are phagocytosed by the macrophages of the vertebrate host. Then, the promastigotes rapidly transform into amastigotes and continue to grow and multiply. When another sand fly sucks blood from an infected host, the amastigote-containing macrophages are transferred and in the sandfly’s midgut the amastigotes are transformed into rapidly dividing log phase promastigotes. In the gut of the sandfly, initially promastigotes rapidly divide which are called logarithmic phase promastigotes (log phase promastigotes) and they have low infectivity. Then, in the gut of the sandfly, promastigotes become non-dividing but more virulent which are called stationary phase promastigotes and ready to infect the host by sandfly bite. Microsoft Office PowerPoint and Paint were used only as a tool during the drawing of the figure and *Phlebotomus* image was obtained from https://commons.wikimedia.org/wiki/File:Phlebotomus_(01).png.

The relatively simple life cycle of *Leishmania* is characterized by two basic phases, the promastigote, the motile form that lives in the gut of the sand fly vector, and the amastigote, the immobile form that resides in vertebrate host macrophages. In the gut of the sandfly, initially promastigotes rapidly divide which are called logarithmic phase promastigotes (log phase promastigotes). At this stage, the parasites exhibit low infectivity. Then, in the gut of the sandfly, promastigotes become non-dividing but more virulent which are called stationary phase promastigotes (Fig 1) [5–7]. During the blood meal, the *Leishmania*-infected sand fly inoculates the stationary phase promastigotes into the skin and the promastigotes are phagocytosed by the macrophages and/or dendritic cells of the vertebrate host. Next, the promastigotes rapidly transform into amastigotes and continue to grow and multiply. When another sand fly sucks blood from an infected host, the amastigote-containing macrophages are transferred and in the sandfly’s midgut the amastigotes are transformed into rapidly dividing log phase promastigotes (Fig 1) [8,9].

*L. infantum*, *L. tropica*, and *L. major* have 36 chromosomes [10]. GP63 protein also known as Leishmanolysin is one of the major surface antigen that facilitates the entry of *Leishmania* parasites into macrophages, contributes to parasite survival and has shown to be immunogenic in limited vaccine studies [11–14]. However, GP63 protein is encoded from different chromosomes and different gene regions (multi-copy gene) in different life stages of *Leishmania* (log phase, stationary phase, amastigote) and there are also differences in its expression between species. Previous studies have reported that one group of genes encoding the GP63 protein: First group (Group 1) is constitutively expressed, where a second group (Group 2) is expressed in the log phase promastigote, and a third group (Group 3) is expressed in the stationary phase promastigote/amastigote [15]. In addition, the metalloprotease GP63 protein contains pro-peptide sequences in the N-terminal and C-terminal regions that are removed during biosynthesis and maturation. These pro-peptide sequences protect the promastigote from the protease activity of the active protein [16]. In this study, we aimed to sequence the more virulent stationary phase promastigote/amastigote specific GP63 gene (Group 3) in *L. infantum*, *L. major* and *L. tropica* isolates (n:59) detected in clinical cases from Türkiye. The sequence data were then used to detect variations in GP63 within each *Leishmania* species. Next, the prevalent GP63 protein sequences in each *Leishmania* species were analyzed by *in silico* analysis to identify novel epitopes to generate a new antigen that could be used in vaccine studies or development of new serological assays.

## Results

### Sample identification

The stationary phase promastigote/amastigote specific GP63 genes in *L. infantum*, *L. major*, and *L. tropica* were amplified by PCR and the ~ 1800 bp amplicons were sequenced. According to the sequence data, similarity rates were changed from 99.78 to 100% for *L. infantum* samples (n: 10), 99.23 to 100% for *L. major* samples (n: 20), and 90.09 to 91.48% for *L. tropica* samples (n: 29) (Table 1). The GP63 gene of *L. infantum* is more conserved than that of *L. major* and *L. tropica*. In contrast, the GP63 gene of *L. tropica* is more polymorphic and the similarity with the reference GP63 gene is reduced to 90.09%. Sequence data were given in S2, S3, and S4 Files.

**Table 1:** BLAST results of GP63 proteins of *L. infantum*, *L. major*, and *L. tropica* samples.

Species	Lab. sample no.	Similarity rate	Accession no	Species	Lab. sample no.	Similarity rate	Accession no
*L. infantum*	60	99.78%	LR812943.1	*L tropica*	3	91.48%	AJ495009.1
	61	99.83%	LR812943.1		4	91.48%	AJ495009.1
	62	99.89%	LR812943.1		5	91.36%	AJ495009.1
	63	100.00%	LR812943.1		6	91.14%	AJ495009.1
	64	99.89%	LR812943.1		8	90.96%	AJ495009.1
	65	99.94%	LR812943.1		9	91.48%	AJ495009.1
	66	99.94%	LR812943.1		11	91.48%	AJ495009.1
	67	99.89%	LR812943.1		13	91.43%	AJ495009.1
	68	99.94%	LR812943.1		14	91.60%	AJ495009.1
	69	99.94%	LR812943.1		16	91.42%	AJ495009.1
*L. major*	39	99.67%	OU755544.1		19	90.09%	AJ495009.1
	40	99.23%	OU755544.1		20	91.14%	AJ495009.1
	42	100.00%	OU755544.1		21	91.13%	AJ495009.1
	43	99.39%	OU755544.1		22	91.30%	AJ495009.1
	44	99.39%	OU755544.1		23	91.36%	AJ495009.1
	45	99.50%	OU755544.1		24	91.54%	AJ495009.1
	46	99.39%	OU755544.1		25	91.42%	AJ495009.1
	47	99.89%	OU755544.1		26	91.37%	AJ495009.1
	48	99.28%	OU755544.1		27	91.37%	AJ495009.1
	49	99.56%	OU755544.1		28	91.48%	AJ495009.1
	50	99.83%	OU755544.1		29	91.37%	AJ495009.1
	51	100.00%	OU755544.1		30	91.48%	AJ495009.1
	52	99.28%	OU755544.1		31	91.48%	AJ495009.1
	53	99.28%	OU755544.1		32	91.42%	AJ495009.1
	54	99.34%	OU755544.1		33	90.90%	AJ495009.1
	55	100.00%	OU755544.1		34	91.14%	AJ495009.1
	56	99.39%	OU755544.1		35	91.42%	AJ495009.1
	57	99.56%	OU755544.1		36	90.79%	AJ495009.1
	58	99.39%	OU755544.1		37	91.48%	AJ495009.1
	59	99.94%	OU755544.1				

### Variations of GP63 proteins

Amino acids sequences of GP63 protein belonging to each sequenced *Leishmania* sample were aligned with reference GP63 proteins (XM_001463664.2, XM_001681327.1, and CM024296.1) using MEGA X. Although GP63 protein has propeptide regions at N-terminal and C-terminal ends, mature GP63 protein presented on the surface of *Leishmania* spp. doesn’t have propeptide regions. Therefore, after removing propeptides of GP63 protein, amino acid region between 101–577 were evaluated and variations were identified.

In *L. infantum* group, five different GP63 variants were identified and named as Variant 1, 2, 3, 4 and 5. Among them only Variant 1 (sample 63) had a 100% similarity with the reference GP63 protein (LR812943.1) (Table 1). However, prevalent GP63 protein was the Variant 3 which is detected in 5 different *L. infantum* samples (samples 65, 66, 67, 68, and 69) (Table 2). For *L. major*, six different GP63 variants were identified and named as Variant 1, 2, 3, 4, 5, and 6. Only Variant 1 was 100% identical to the reference GP63 protein (OU755544.1) (Table 1) and at the same time, prevalent GP63 protein was also Variant 1 which is identical to reference GP63 protein detected in 8 different *L. major* samples (Table 3). When compared to the *L. infantum* and *L. major*, more variations were identified in GP63 protein of *L. tropica* samples. For *L. tropica*, 19 different GP63 variants were identified and named as Variant 1, 2, 3…, and 19. Any *L. tropica* isolate did not show %100 similarity with the reference GP63 protein (AJ495009.1) (Table 1). Among them, Variant 1 was most identical to the reference GP63 protein and at the same time, prevalent GP63 protein was also Variant 1 and detected in 9 different *L. tropica* samples (Table 4).

**Table 2:** Variation analysis performed for *L. infantum* samples using reference GP63 protein (XM_001463664.2).

No of *L. infantum* samples	Variants detected	Amino acid variation positions of GP63 protein			
		368	390	396	409
		D	C	T	T
63	Variant 1	*	*	*	*
62, 64	Variant 2	N	*	*	A
65, 66, 67, 68, 69	Variant 3	*	*	*	A
60, 61	Variant 4	*	G	P	A

**Table 3:** Variation analysis performed for *L. major* samples using reference GP63 protein (XM_001681327.1).

No of *L. major* samples	Variants detected	Amino acid variation positions of GP63 protein								
		169	206	275	278	285	362	397	398	448
		T	S	P	E	S	L	N	I	V
39, 40, 42, 47, 50, 51, 55, 59	Variant 1	*	*	*	*	*	*	*	*	*
43, 53, 54, 56, 58	Variant 2	K	G	L	K	N	F	S	V	L
44, 46, 48	Variant 3	K	G	L	K	N	F	*	V	L
52, 57	Variant 4	K	G	*	*	N	F	S	V	*
45	Variant 5	*	*	L	R	N	F	*	*	*
49	Variant 6	K	*	L	K	N	*	S	V	L

**Table 4:** Variation analysis performed for *L. tropica* samples using reference GP63 protein (CM024296.1).

No of *L. tropica* samples	Variants detected	Amino acid variation positions of GP63 protein															
110	111	136	166	189	236	244	257	280	293	327	336	396	398	458	497
R	I	A	Q	E	G	N	A	A	G	G	K	D	T	V	T
3, 4, 9, 11, 16, 28, 30, 31, 37	Variant 1	*	V	*	*	*	*	*	*	*	*	S	*	*	*	*	A
5, 32	Variant 2	*	V	*	*	*	*	*	*	*	*	S	*	*	*	*	*
6	Variant 3	*	*	*	L	D	D	I	D	D	D	*	*	*	*	E	A
8	Variant 4	L	*	D	L	D	D	*	D	*	*	*	*	*	*	*	*
13	Variant 5	*	*	*	*	*	*	*	D	*	D	*	M	*	I	E	*
14	Variant 6	*	*	*	*	*	*	I	D	*	D	*	M	Q	I	E	*
19	Variant 7	L	*	D	*	*	*	*	*	*	*	*	*	*	*	E	A
20	Variant 8	L	V	D	*	*	*	*	*	*	*	*	*	*	*	E	*
21	Variant 9	*	V	D	*	*	*	I	D	*	D	*	M	Q	*	*	*
22	Variant 10	*	*	*	*	*	*	*	*	*	*	S	*	*	*	*	A
23	Variant 11	*	*	*	*	*	*	*	*	*	*	S	*	*	*	*	A
24	Variant 12	*	*	*	*	*	*	*	D	*	D	*	*	Q	I	E	*
25	Variant 13	L	*	*	*	*	*	*	D	*	D	*	M	Q	I	*	A
26	Variant 14	*	*	*	*	*	*	*	D	*	D	*	M	Q	*	E	*
27, 29	Variant 15	*	*	*	*	*	*	*	D	*	D	*	M	*	*	E	*
33	Variant 16	*	*	*	L	D	*	*	D	D	D	*	M	*	*	E	A
34	Variant 17	*	*	*	*	*	*	*	D	*	*	*	*	*	*	E	*
35	Variant 18	*	*	*	*	*	*	*	*	*	*	S	*	*	*	*	A
36	Variant 19	L	*	D	L	D	D	I	D	*	*	*	*	*	*	E	A

### Properties of selected GP63 proteins

When selected GP63 proteins were compared, the number of amino acids were 477 for *L. infantum* and *L. major* (except of *L. tropica* which was 476 aa) and molecular weights were ~51.5 kDa. The theoretical *p*I values were between 5.23 and 5.83. All proteins had a half-life of 30 hours and were stable. Aliphatic indexes were between 71.64 and 75.68. For the selected GP63 proteins, the alpha helix scores were between 23.06% and 27.67%, the extended strand scores were between 19.08% and 23.90%, and the random coil scores were between 50.94% and 53.25% (Table 7). All the selected GP63 proteins were antigenic and non-allergenic. The GP63 proteins selected for *L. infantum* and *L. major* have N-glycosylation sites and the GP63 proteins selected for *L. infantum*, *L. major*, and *L. tropica* have O-glycosylation sites. (Table 6).

**Table 5:** Physico-chemical parameters of selected GP63 proteins.

*Leishmania *species	Number of amino acids	Molecular weight (Da)	Therotical *p*I	Estimated half-life (h)	Instability index (II)	Aliphatic index	GRAVY	Alpha helix (%)	Extended strand (%)	Random coil (%)
*L. infantum* GP63	477	51641.29	5.83	30	39.73	71.64	-0.186	27.67	19.08	53.25
*L. major* GP63	477	51549.06	5.23	30	36.00	75.68	-0.111	23.06	23.90	53.04
*L. tropica* GP63	476	51673.17	5.55	30	37.04	71.69	-0.146	26.21	22.85	50.94

**Table 6:** Antigenicity, allergenicity, post-translational modification predictions of selected GP63 proteins.

*Leishmania *species	Antigenicity	Allergenicity	N-Glycosylation position and motif	O-Glycosylation position
*L. infantum* GP63	0.5901	Non-Allergen	297, NITK	14, 33, 34, 37, 40, 187, 194, 328, 371
*L. major* GP63	0.5887	Non-Allergen	297, NITQ 307, NESE	14, 328
*L. tropica* GP63	0.5708	Non-Allergen	**–**	14, 298, 316, 323, 327

**Table 7:** B cell epitope predictions in selected GP63 proteins.

*Leishmania *species	Epitopes	Antigenicity	Toxicity	Allergenicity	Solubility	Heavy chain	Light chain
*L. infantum* GP63	EDILTDEKRDILVKH	1.1605	Non-toxin	Non-allergen	Good water solubility	Score: -183.13	Score: -169.57
	
	LQLHTERLKVRQVQDKWKVT	0.7839	Non-toxin	Non-allergen	Good water solubility	Score: -191.71	Score: -220.12
	
	**GSHIK**MRNAQDEL*	0.5164	Non-toxin	Non-allergen	Good water solubility	Score: -193.14	Score: -193.39
	
	**DGAFRPKTS**HG*	0.5293	Non-toxin	Non-allergen	Good water solubility	Score: -183.69	Score: -164.91
	
*L. major* GP63	QLHTERLKVQQVQGKWKVT	0.5300	Non-toxin	Non-allergen	Good water solubility	Score: -197.80	Score: -197.09
	
	EMEDQGSAGSAGS	2.2086	Non-toxin	Non-allergen	Good water solubility	Score: -123.35	Score: -131.88
	
	**SHIKMRN**AQDEL*	0.5026	Non-toxin	Non-allergen	Good water solubility	Score: -180.62	Score: -189.69
	
*L. tropica* GP63	EDILTDEKRDILRKY	0.6688	Non-toxin	Non-allergen	Good water solubility	Score: -185.98	Score: -164.95
	
	LQLHTERLKARQVQGKWKVT	0.9818	Non-toxin	Non-allergen	Good water solubility	Score: -189.27	Score: -184.34
	
	**SHIKMRN**AQDEL*	0.5026	Non-toxin	Non-allergen	Good water solubility	Score: -180.62	Score: -189.69
	

*Bold indicates that the amino acid residues also were detected as non-linear B cell epitope.

### B-cell epitopes

Several B cell epitopes were predicted in the GP63 protein of *L. infantum*, *L. major*, and *L. tropica*. When an epitope was antigenic, non-toxic, non-allergenic, and soluble, it was selected as a B cell epitope. Among these B cell epitopes, the EMEDQGSAGSAGSAGS epitope predicted in GP63 protein of *L. major* had the highest antigenicity value (2.2086). In the GP63 protein of *L. infantum*, EDILTDEKRDILVKH epitope had the highest antigenicity value (1.1605), while the LQLHTERLKARQVQGKWKVT epitope predicted on the GP63 protein of *L. tropica* had the highest antigenicity value (0.9818). Interestingly, these epitopes with high antigenicity values did not have residue/s presented in non-linear B cell epitope. An epitope LQLHTERLKARQVQGKWKVT was found in all selected GP63 protein with minor variations in some residues (underlined residues). Another epitope, EDILTDEKRDILVKH, was found in selected GP63 proteins of *L. infantum* and *L. tropica* with minor variations in some residues (underlined residues) and these two amino acids variation caused a dramatic change in their antigenicity.

A different epitope, **SHIKMRN**AQDEL, shared by selected GP63 proteins of *L. major* and *L. tropica* has residues presented in the non-linear B cell epitope (bold residues). Moreover, epitopes with residue(s) presented in the non-linear B cell epitope had lower antigenicity values (Table 7). According to the docking results, all B cell epitopes were docked to variable regions of human B cell heavy and light chains of Fab fragment.

### MHC-I/II epitopes

Several MHC-I/II epitopes were predicted in the GP63 protein of *L. infantum*, *L. major*, and *L. tropica*. When an epitope was antigenic, non-toxic, non-allergenic and soluble, it was selected as an MHC-I or MHC-II epitope. Among the MHC-I epitopes, the STHDSGSTTC epitope predicted in GP63 protein of *L. infantum* had the highest antigenicity value (1.5063) (Table 8). The RLKVQQVQGK epitope predicted on the GP63 protein of *L. major* had the highest antigenicity value (1.0311), while no epitope was predicted on the GP63 protein of *L. tropica*. The predicted epitopes were non-toxic, non-allergenic, and soluble (Table 9). An epitope, RLKVRQVQDK, was predicted in selected GP63 proteins of *L. infantum* and *L. major* with minor changes in some residues (underlined residues) and these two amino acid changes caused slight change in antigenicity. Another epitope, KRDILVKHLI, was shared with GP63 proteins of *L. infantum* and *L. major*.

**Table 8:** MHC-I epitope predictions in selected GP63 proteins of *L. infantum.*

Allele	Epitope	Peptide score	Percentile rank	Antigenicity	Toxicity	Allergenicity	Solubility
HLA-B*27:05	SRYNQLVTRV	0.590111	0.23	0.7910	Non-toxin	Non-allergen	Good water solubility
HLA-A*03:01	RLKVRQVQDK	0.523148	0.31	1.2412	Non-toxin	Non-allergen	Good water solubility
HLA-B*40:01	SEAGAPFKGF	0.436832	0.32	0.5369	Non-toxin	Non-allergen	Good water solubility
HLA-B*27:05	RRKTSKVPVL	0.432353	0.43	0.5238	Non-toxin	Non-allergen	Good water solubility
HLA-A*03:01	RASEAGAPFK	0.416625	0.46	0.7832	Non-toxin	Non-allergen	Good water solubility
HLA-B*27:05; HLA-A*01:01; HLA-A*30:02	VRCDTATRTY	0.40479	0.46	0.7038	Non-toxin	Non-allergen	Good water solubility
HLA-A*03:01; HLA-B*57:01	KVRQVQDKWK	0.379627	0.52	0.5925	Non-toxin	Non-allergen	Good water solubility
HLA-B*27:05	ARVGQRISTH	0.287762	0.69	0.8027	Non-toxin	Non-allergen	Good water solubility
HLA-B*27:05	KRDILVKHLI	0.264775	0.73	0.8167	Non-toxin	Non-allergen	Good water solubility
HLA-A*01:01; HLA-A*30:02	VSSAFEEGGY	0.210287	0.52	1.0543	Non-toxin	Non-allergen	Good water solubility
HLA-B*27:05	LRIAVSTEDL	0.194423	0.94	0.8171	Non-toxin	Non-allergen	Good water solubility
HLA-B*39:01	STHDSGSTTC	0.130252	0.75	1.5063	Non-toxin	Non-allergen	Good water solubility
HLA-B*39:01	YHCARVGQRI	0.100039	0.92	0.8593	Non-toxin	Non-allergen	Good water solubility

**Table 9:** MHC-I epitope predictions in selected GP63 protein of *L. major.*

Allele	Epitope	Peptide score	Percentile rank	Antigenicity	Toxicity	Allergenicity	Solubility
HLA-A*03:01	RLKVQQVQGK	0.802882	0.08	1.0311	Non-toxin	Non-allergen	Good water solubility
HLA-B*27:05	SRYDQLVTRV	0.734979	0.12	0.6061	Non-toxin	Non-allergen	Good water solubility
HLA-B*27:05	IRCPTSRLSL	0.34089	0.57	0.5890	Non-toxin	Non-allergen	Good water solubility
HLA-B*27:05	KRDILVKHLI	0.264775	0.73	0.8167	Non-toxin	Non-allergen	Good water solubility
HLA-A*03:01	RIVASVPNVR	0.264049	0.74	0.9084	Non-toxin	Non-allergen	Good water solubility

**Fig 2:**
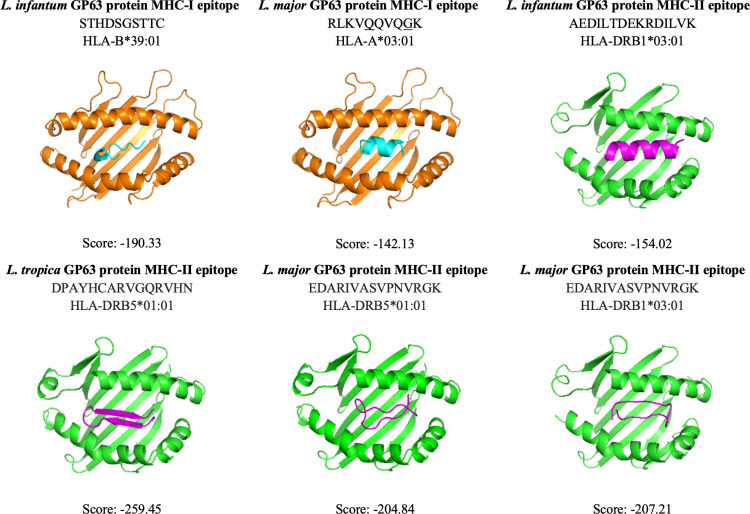
Docking results of selected MHC-I/II epitopes.

**Fig 3:**
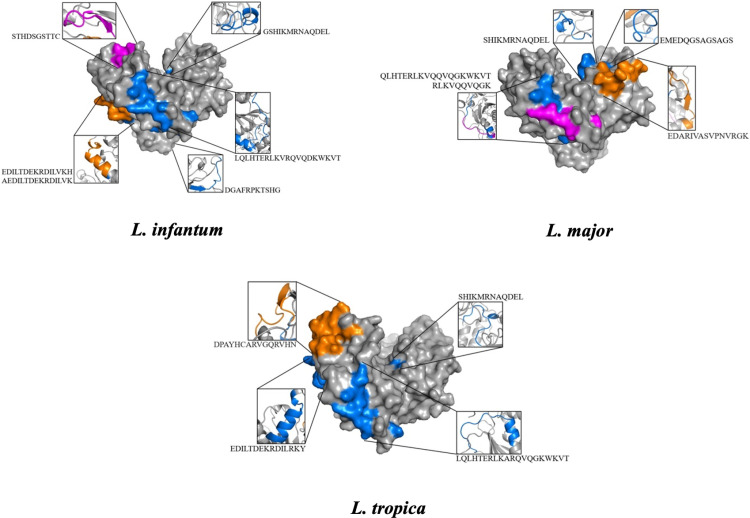
3D structures of selected GRP63 proteins, showing epitopes selected and docked with BCR or MHC-I/II alleles.

Among the MHC-II epitopes, the AEDILTDEKRDILVK epitope predicted on GP63 protein of *L. infantum* had the highest antigenicity value (1.1978) (Table 10). The EDARIVASVPNVRGK epitope predicted on the GP63 protein of *L. major* had the highest antigenicity value (1.180), while the DPAYHCARVGQRVHN epitope predicted on the GP63 protein of *L. tropica* had the highest antigenicity value (1.1654) (Tables 11 and 12). An epitope, CTAEDILTDEKRDIL was shared with selected GP63 proteins of *L. infantum* and *L. tropica*. Also, four epitopes (AEDILTDEKRDILRK, TAEDILTDEKRDILR, EDILTDEKRDILRKY, and DPAYHCARVGQRVHN) were detected in selected GP63 proteins of *L. infantum* and *L. tropica* with minor changes in some residues (underlined residues) and these two amino acid changes caused dramatic changes in antigenicity values. According to the docking results, MHC-I/II epitopes with highest antigenicity value were docked to their MHC-I/II alleles with a different docking score (Figs 2 and 3).

**Table 10:** MHC-II epitope predictions in selected GP63 proteins of *L. infantum.*

Allele	Epitope	Peptide score	Percentile rank	Antigenicity	Toxicity	Allergenicity	Solubility
HLA-DRB1*03:01	AEDILTDEKRDILVK	0.9519	0.1	1.1978	Non-toxin	Non-allergen	Good water solubility
HLA-DRB1*03:01	TAEDILTDEKRDILV	0.9285	0.23	0.9852	Non-toxin	Non-allergen	Good water solubility
HLA-DRB1*03:01	CTAEDILTDEKRDIL	0.8898	0.37	0.7248	Non-toxin	Non-allergen	Good water solubility
HLA-DRB1*03:01	EDILTDEKRDILVKH	0.8758	0.45	1.1605	Non-toxin	Non-allergen	Good water solubility
HLA-DRB5*01:01	DPAYHCARVGQRIST	0.5990	0.62	0.9469	Non-toxin	Non-allergen	Good water solubility
HLA-DRB4*01:01	KHLIPQALQLHTERL	0.4703	0.85	0.6541	Non-toxin	Non-allergen	Good water solubility

**Table 11:** MHC-II epitope predictions in selected GP63 proteins of *L. major.*

Allele	Peptide	Peptide score	Percentile rank	Antigenicity	Toxicity	Allergenicity	Solubility
HLA-DRB5*01:01; HLA-DRB1*03:01; HLA-DRB3*02:02	DARIVASVPNVRGKN	0.7603	0.21	0.9743	Non-toxin	Non-allergen	Good water solubility
HLA-DRB5*01:01; HLA-DRB1*03:01	EDARIVASVPNVRGK	0.7011	0.31	1.1180	Non-toxin	Non-allergen	Good water solubility

**Table 12:** MHC-II epitope predictions in selected GP63 proteins of *L. tropica.*

Allele	Peptide	Peptide score	Percentile rank	Antigenicity	Toxicity	Allergenicity	Solubility
HLA-DRB1*03:01	AEDILTDEKRDILRK	0.9628	0.07	0.7265	Non-toxin	Non-allergen	Good water solubility
HLA-DRB1*03:01	TAEDILTDEKRDILR	0.9468	0.12	0.6699	Non-toxin	Non-allergen	Good water solubility
HLA-DRB4*01:01	RKYLIPQALQLHTER	0.6653	0.32	0.5670	Non-toxin	Non-allergen	Good water solubility
HLA-DRB1*03:01	EDILTDEKRDILRKY	0.9075	0.33	0.6688	Non-toxin	Non-allergen	Good water solubility
HLA-DRB1*03:01	CTAEDILTDEKRDIL	0.8898	0.37	0.7248	Non-toxin	Non-allergen	Good water solubility
HLA-DRB5*01:01	DPAYHCARVGQRVHN	0.6625	0.40	1.1654	Non-toxin	Non-allergen	Good water solubility

## Discussion

There are three main clinical forms of leishmaniasis: cutaneous leishmaniasis (CL), visceral leishmaniasis (VL), and mucocutaneous leishmaniasis (MCL), each associated with specific clinical presentation. Although leishmaniasis has devastating effects and is the most common parasitic disease after malaria, prevention and control of this disease is challenging due to the difficulty of the treatment course and the lack of a safe vaccine that confers complete protection [17]. Among ongoing vaccine studies, only ChAd63-KH completed phase2b clinical trials in 2023 [18]. Developing a vaccine for leishmaniasis is challenging due to complex life cycle of *Leishmania* species. During its life cycle, many proteins are involved in entry and egress from the host cell as well as in evading the immune response. Among these proteins, the GP63 surface protein allows the parasite to adhere to host macrophages and escape phagocytosis. In addition, GP63 has been used as antigen in different vaccine studies due to its important role in the pathogenesis of *Leishmania* species. For example, in a study, mice were vaccinated by CpG ODN co-encapsulated with recombinant GP63 protein in cationic liposomes and it achieved reduction in spleen parasite burden, higher IgG2a/IgG1 ratio, IFN-γ levels, and lower IL-4 levels compared to other groups [12]. In another study, after GP63 encoding gene was introduced into the *Salmonella typhimurium* which is a live oral vaccine strain, mice were vaccinated with this *Salmonella typhimurium*. The results showed that a significant resistance developed against challenge with *L. major* compared to controls [11]. In a different study, three groups of mice were initially vaccinated by DNA vaccines encoding GP63, Kmp-11, and Amastin proteins followed by booster with recombinant GP63, Kmp-11, and Amastin proteins. According to the challenging results of mice which were intraperitoneally administered with *L. infantum*, splenic parasitic loads decreased by 89%, 86%, and 79% in GP63, Kmp-11, and Amastin vaccine groups, respectively [14].

Although the GP63 protein has been used as an antigen in a limited number of vaccine studies, it is not clear whether cross-species variations in the *Leishmania* spp. multi-copy GP63 gene have been taken into account or which copy of GP63 was used. Previous studies have reported that the stationary phase promastigote/amastigote form is highly virulent and responsible for human infection [15]. Thus, GP63 expressed in stationary phase promastigote and consequently in amastigote in the host, is an important vaccine target.

To analyze this issue more in depth, we first sequenced the GP63 gene expressed in the stationary phase promastigote/amastigote form in *L. infantum*, *L. major*, and *L. tropica* isolates detected from clinical cases in Türkiye. According to the results, many variations were detected in the GP63 protein expressed in the stationary phase promastigote/amastigote form in *L. infantum*, *L. major*, and *L. tropica* isolates. Among *Leishmania* species, the stationary phase promastigote/amastigote GP63 protein of *L. tropica* showed noticeably more variation compared to *L. major* and *L. infantum* as shown by BLAST similarity results which was between 90.09-91.48% for *L. tropica* isolates, 99.78 to 100% for *L. infantum* isolates, and 99.23 to 100% for *L. major* isolates (Table 1). These data indicate that the GP63 protein derived from stationary-phase promastigotes/amastigotes of *Leishmania infantum*, a causative agent of visceral leishmaniasis, exhibits a higher degree of sequence conservation compared to the corresponding GP63 proteins from *L. tropica* and *L. major*, which are associated with cutaneous leishmaniasis. This may be due to narrower range of *Phlebotomus* vector, mammalians hosts, restricted distribution of *L. infantum* compared to *L. tropica* and *L. major* resulting with a more conserved genome. Moreover, while *L. infantum* is primarily zoonotic, *L. tropica* is more frequently acquired through anthroponotic transmission, and *L. major* has numerous wild rodent reservoirs, which may result in increased evolutionary pressures and genomic plasticity. It has been showed that the average amino acid identity between *L. major* and *L. infantum* was 92%, and the average nucleotide identity was 94% which indicates that they both have very similar genomes, both at the DNA and protein levels [19] and supports our results in which *L. infantum* and *L. major* have more conserved GP63 proteins compared to *L. tropica* (Tables 4, 5, and 6)*.* Mosaic aneuploidy detected in different levels in various *Leishmania spp.* can also be linked with this GP63 variability between Leishmania isolates from different strains [20]. The GP63 protein variation data was important as it showed that if the GP63 protein of *L. tropica* is used as an antigen in any vaccine study, variation analysis has utmost importance for the selection of vaccine antigen.

There isn’t any significant difference between the selected GP63 proteins in terms of antigenicity value, allergenicity, post translational modifications, and physico-chemical parameters. However, the GRAVY value of *L. infantum* GP63 protein was lower than that of other species, which points to a higher degree of hydrophilicity. This indicates that *L. infantum* GP63 protein interacts more positively with surrounding water molecules, which may increase its solubility [21]. Such a property is especially advantageous for recombinant protein production, because the increased hydrophilicity facilitates higher expression levels and improved folding efficiency in expression systems. On the other hand, the GP63 protein of *L. major* showed a higher aliphatic index, which indicates greater thermal stability over a wide temperature range while the GP63 protein of *L. infantum* showed a higher proportion of random coil structures, which may increase its structural flexibility and potentially increase its accessibility for antibody recognition [21,22]. Interestingly, while the GP63 proteins of *L. infantum* and *L. major* contained both N- and O-glycosylation motifs, the GP63 protein of *L. tropica* had only O-glycosylation sites and lacked any detectable N-glycosylation motifs. The absence of N-linked glycosylation sites in *L. tropica* could be attributed to the higher degree of sequence variation observed in the GP63 protein, which could disrupt the consensus sequences required for N-glycosylation. This feature also suggests that the GP63 protein of *L. tropica* could potentially be expressed in prokaryotic expression systems that are more cost-effective and technically simpler than eukaryotic platforms [23,24]. All predicted instability index were below the threshold value of 40, indicating predicted *in vitro* stability. However, the value for the *L. infantum* GP63 protein was closer to the threshold, suggesting that it may be slightly less stable than the others and potentially more sensitive to environmental conditions such as temperature or pH. When these parameters are evaluated collectively, the GP63 protein of *L. infantum* appears to be a more promising candidate for vaccine development due to its higher hydrophilicity, increased random coil structure, and the presence of post-translational modifications. Although its predicted stability is slightly lower than others, its overall biochemical and structural features offer advantages for recombinant production and immunogenic potential.

Secondly, the GP63 protein, which was consistently found to be highly represented across all analyzed *Leishmania* species, was selected for subsequent epitope prediction analyses. Several B cell epitopes were predicted in the GP63 protein of *L. infantum*, *L. major*, and *L. tropica*. All epitopes selected in this study are antigenic and soluble, as well as non-toxic and non-allergenic. Thus, all of them can be used as antigens in vaccine studies and serological tests such as ELISA or LFA (Lateral Flow Assay). Among these B cell epitopes, the EMEDQGSAGSAGSAGS epitope predicted on GP63 protein of *L. major* had the highest antigenicity value (2.2086). The EDILTDEKRDILVKH epitope predicted on the GP63 protein of *L. infantum* had the highest antigenicity value (1.1605), while the LQLHTERLKARQVQGKWKVT epitope predicted on the GP63 protein of *L. tropica* had the highest antigenicity value (0.9818). An epitope LQLHTERLKARQVQGKWKVT was conserved in all selected GP63 protein with a minor variations in some residues (underlined residues). In addition, EDILTDEKRDILVKH (underlined residues shows minor changes) epitope was conserved among *L. infantum* and *L. tropica* whereas SHIKMRNAQDEL epitope was conserved among *L. major* and *L. tropica*. These results suggest that these conserved epitopes could be used in development of the serological test targeting all three *Leishmania* species or two *Leishmania* species. Similarly, conserved MHC-I (RLKVRQVQDK, KRDILVKHLI for *L. infantum* and *L. major*; underlined residues show minor variations) and MHC-II epitopes (CTAEDILTDEKRDIL, AEDILTDEKRDILRK, TAEDILTDEKRDILR, EDILTDEKRDILRKY and DPAYHCARVGQRVHN for *L. infantum* and *L. tropica*; underlined residues show minor variations) were also identified. These results likewise suggest that such conserved epitopes could be used in epitope vaccine studies targeting two different *Leishmania* species. Moreover, it was proposed that the incorporation of conserved B cell epitopes into a multi-epitope vaccine construct could enhance the humoral immune response complementing the cellular immune response elicited by inclusion of conserved MHC-I/II T cell epitopes.

## Conclusion

GP63 gene expressed in the stationary phase promastigote/amastigote form in *L. infantum*, *L. major*, and *L. tropica* isolates detected in clinical cases from Türkiye was sequenced for the first time. During variation analysis, the most common GP63 protein variants for each *Leishmania* species were identified and analyzed for epitope discovery using an *in silico* approach. Although a large number of epitopes were predicted, some were promising due to their high antigenicity values or conservation among *Leishmania* species. Overall, these promising epitopes could be used as antigens in vaccine studies and in the development of new serologic diagnostic assays.

## Materials and methods

### Ethics statement

This study was approved by the Research Ethics Committee of the Ege University Faculty of Medicine (Approval number: 24-6.1T/54) for the use of DNA samples. Written informed consent was provided to all patients, data privacy protection was guaranteed by anonymization of DNA samples.

### DNA samples

*L. tropica* (n = 29), *L. major* (n = 20), *L. infantum* (n = 10) isolates used in this study were isolated from different clinical cases that were admitted to Celal Bayar University Hospital in Manisa province of Türkiye and were cryopreserved. DNA was extracted from these samples by a commercial DNA isolation kit (Zymo Research) and used in PCR studies. The *Leishmania* isolates analyzed were in this study were named as MHOM/TR/Year/Laboratory Number. Clinical characteristics of the patients from whom the parasites were isolated are provided in S1 File.

### Selection of stationary phase promastigote/amastigote specific GP63 genes and primer design

The stationary phase promastigote/amastigote specific GP63 gene region was targeted during PCR. The stationary phase promastigote/amastigote-specific GP63 gene sequences of *L. tropica* and *L. infantum* were obtained from TriTryDB (https://tritrypdb.org/) database. In a previous study by Castro Neto et al. (2019), accession numbers LinJ.10.0520 and LmjF.10.0480 of *L. infantum* (GenBank: XM_001463664.2) and *L. major* (GenBank:XM_001681327.1) were reported to correspond to stationary phase/amastigote-specific GP63, respectively [15]. However, there is not any information about *L. tropica*. For this reason, the stationary phase promastigote/amastigote-specific GP63 gene of *L. major* was used as a template to find that of *L. tropica*, since existing molecular phylogenetic studies show that *L. tropica* and *L. major* are more closely related to each other, while *L. infantum* is in a more distant phylogenetic position than these two species. During analysis, *L. major* GP63 gene sequence (LmjF.10.0480) was blasted in the NCBI by selecting *L. tropica* as organism and the GP63 sequence with highest similarity rate (genome accession number in GenBank: CM024296.1) was accepted as the stationary phase promastigote/amastigote-specific GP63 gene. GP63 is a multi-copy gene and GP63 gene regions within a *Leishmania* species or between *Leishmania* species are similar to each other. Therefore, primers designed from the exon sequence annealed to different GP63 sites and generated multiple band patterns during PCR that were rendered unacceptable for sequencing. To solve this problem, forward and reverse primers were designed from the GP63 gene 5’UTR and 3’UTR intron regions of each Leishmania species as well as two additional sequencing primers using the NCBI Primer design tool (https://www.ncbi.nlm.nih.gov/tools/primer-blast/) (Table 13).

**Table 13:** Primers used for amplification and sequencing of selected GR63 gene intron regions of *Leishmania* spp.

Species and GenBank Accession no	PCR primers	PCR product size	Sequencing primers
*L. infantum* (GenBank: XM_001463664.2)	LiF: 5’-TCTTTCTTTTCCGTCGCTTC-3’ LiR: 5’-AGCTCTTCCGAGAGACAAC-3’	1800 bp	LiF1: 5’-CACCCCACTGCCCACAG-3’ LiF3: 5’-AGCGAGAAGTGCATGGAGC-3’
*L. major* (GenBank: XM_001681327.1)	LmF: 5’-TGCAAGCGTCATTGATACAC-3’ LmR: 5’-GAGCTCTTCCGAAAGACAATG-3’	1809 bp	Lm-F1: 5’-ATGTCCGTGGACAGCAGC-3’ Lm-R2: 5’-TAACACCGCATGCACCGAG-3’
*L. tropica* (GenBank: CM024296.1)	LtF:5′-ATGCCGCCGAACTAGGTGCACGTCGTCGGCACGAC-3′ LtR: 5′-AAGGGACAGCGTGGAGCTCTTCCGAGAGACAACGG-3′	1818 bp	LtF1: 5′-CTGCAGAGCCATGTCCGT-3’ LtR2: 5′-TGGCCGTGTCGCACC-3’

### PCR and sequencing

PCR reaction for *L. tropica* in final volume of 50 μl included 1.5 μl template DNA, 10 µl 5x SuperFi II Buffer, 1 µl SuperFi II Taq Polimerase, 1 µl dNTPs, 0.05 µM of each primer and 36 μl distilled water. The following PCR cycle was used: 30 sec initial denaturation at 98 ºC, followed by 35 cycles of 10 sec denaturation at 98 ºC, 2 min extension at 72 ºC with a 5 min final extension at 72 ºC.

PCR reaction for *L. major* and *L. infantum* in final volume of 50 μl included 0.5 μl template DNA, 10 µl 5x SuperFi II Buffer, 1 µl Superfii Taq Polimerase, 1 µl dNTPs, 0.1 µM of each primer and 36.5 μl distilled water. The following PCR cycle was used: 30 sec initial denaturation at 98 ºC, followed by 35 cycles of 10 sec denaturation at 98 ºC, 10 sec annealing at 60 ºC, 2 min extension at 72 ºC with a 5 min final extension at 72 ºC. After amplification, PCR products were visualized on a 1% agarose gel and sequenced by the PCR primers as well as two additional primers defined for sequencing in Table 1 using the ABI 3740xl DNA Sequencer (Applied Biosystems). The sequence data obtained from each *Leishmania* species were aligned and translated to amino acid sequence by MEGA X [25] and variations in the stationary phase promastigote/amastigote specific GP63 protein were detected. The prevalent stationary phase promastigote/amastigote specific GP63 proteins within each *Leishmania* species were used in further *in silico* predictions.

### Bioinformatics analyses of the selected GP63 proteins

During bioinformatic analyses, psychochemical properties, secondary structure, allergenicity, antigenicity, N-glycosylation and O-glycosylation sites, 3D structure, toxicity, and solubility of selected GP63 proteins were analyzed by tools described in Table 14.

**Table 14:** Bioinformatics analyses tools used to analyze GP63 protein and its epitopes.

Bioinformatic analyses	Tools used	Link	References
Psychochemical properties	Expasy ProtParam	https://web.expasy.org/protparam/	[26]
Secondary structure prediction	GOR IV	https://npsa-pbil.ibcp.fr/cgi-bin/npsa_automat.pl?page=/NPSA/npsa_gor4.html	[27]
Allergenicity	AllerTOP	https://www.ddg-pharmfac.net/AllerTOP/index.html	[28]
	AlgPred v2.0*	https://webs.iiitd.edu.in/raghava/algpred2/algo.html	[29]
Antigenicity	Vaxijen v2.0	http://www.ddg-pharmfac.net/vaxijen/VaxiJen/VaxiJen.html	[30]
N-glycosylation	NetNGlyc 1.0	https://services.healthtech.dtu.dk/services/NetNGlyc-1.0/	[31]
O-glycosylation	NetOGlyc 4.0	https://services.healthtech.dtu.dk/services/NetOGlyc-4.0/	[32]
3D structure	Alphafold 3	https://alphafoldserver.com/	[33]
Toxicity	ToxinPred*	https://webs.iiitd.edu.in/raghava/toxinpred/protein.php	[34]
Solubility	PepCalc*	https://pepcalc.com/	[35]

*indicates the tool used for peptide analysis.

### Immunoinformatic analyses of the selected GP63 proteins

#### B-cell epitope prediction.

Linear B cell epitopes of selected GP63 proteins were predicted by Bcepred online server (http://crdd.osdd.net/raghava/bcepred/) [36]. Hydrophilicity, flexibility, accessibility, turns, exposed surface, polarity, and antigenic propensity parameters were selected as default and epitopes that have at least three of these parameters were selected for solubility, antigenicity, toxicity, and allergenicity predictions. Non-allergenic, non-toxic, soluble, and antigenic epitopes were accepted as B cell epitopes. Also, non-linear B cell epitopes of selected GP63 proteins were predicted by Ellipro online server (http://tools.iedb.org/ellipro/) [37] and residues detected as non-linear epitopes were shown on linear B cell epitopes.

#### MHC-I and MHC-II epitope prediction.

The prediction of MHC-I and MHC-II epitopes of the selected GP63 proteins were analyzed by NetMHCpan 4.1 EL method [38] using IEDB tool (https://www.iedb.org/). For the prediction of MHC-I epitopes, twelve different HLA supertypes (A01.01, A02.01, A03.01, A24.02, A26.01, B07.02, B08.01, B27. 05, B39.01, B40.01, B58. 01 and B15.01) and 9 different HLA alleles (A02.06, A23.01, A30.02, A32.01, B51.01, B57.01, A68.02, B35.01 and B53.01) proposed for *L. donovani* in a previous study were used [39]. Additionally, for MHC-II epitope prediction, seven different MHC-II alleles (DRB1.03.01, DRB1.07.01, DRB1.15.01, DRB3.01.01, DRB3.02.02, DRB4.01.01, and DRB5.01.01) were used during the analysis. Non-allergenic, non-toxic, soluble, and antigenic epitopes were accepted as MHC-I/II epitopes.

#### Docking analysis.

A three-dimensional structure of the selected peptides was generated using PEP-FOLD 3 (https://bioserv.rpbs.univ-paris-diderot.fr/services/PEP-FOLD3/) [40]. All selected B cell epitopes were docked with human B cell heavy and light chain of Fab fragment (PDB: 5DRW). Also, for each selected GP63 protein of *L. infantum, L. major*, and *L. tropica*, MHC-I or MHC-II epitopes with highest antigenicity value were docked with their MHC-I (PDB: 8RRO for HLA-A*03:01 and PDB: 4O2E for HLA-B*39:01) and MHC-II (PDB: Q30154 for HLA-DRB5*01:01 and 3D structure of HLA-DRB1*03:01 predicted with AlphaFold3 and HDOCK) alleles using the HDOCK (http://hdock.phys.hust.edu.cn/) [41] and visualized in the Pymol (https://www.pymol.org/) [42].

## Supporting information

S1 File Clinical characteristics of the patients from whom the parasites were isolated. (XLSX)

S2 File Sequence data belonging to *L. infantum.* (DOCX)

S3 File Sequence data belonging to *L. major.* (DOCX)

S4 File Sequence data belonging to *L. tropica.* (DOCX)
