# Supplementary material for: In silico identification of Leishmania GP63 protein epitopes to generate a new vaccine antigen against leishmaniasis
Source: PLoS Negl Trop Dis. 2025 Jun 5;19(6):e0013137. doi: 10.1371/journal.pntd.0013137 (PMC12140206; doi:10.1371/journal.pntd.0013137)
Supplement: S2 File — (DOCX) [file pntd.0013137.s002.docx]

L. infantum

>Variant_1

ATGTCCGTCGACAGCAGCAGCACGCACCGGCACCGCAGCGTCGCCGCGCGCCTGGTGCGCCTCGCGGCTGCCGGCGCCGCAGTCATCGCTGCTGTCGGCACCGCGGCCGCGTGGGCACACGCCGGTGCGGTGCAGCACCGCTGCATCCACGACGCGATGCAGGCACGCGTGCGGCAGTCGGTGGCGCGCCACCACACGGCCCCCGGCGCCGTGTCCGCGGTGGGCCTGCCGTACGTTACTCTCGACACCGCGGCCGCCGCCGATCGCCGGCCGGGCAGCGCGCCTACAGTCGTGCGCGCCGCGAACTGGGGCGCGCTGCGCATCGCCGTCTCCACCGAGGACCTCACCGACCCCGCCTACCACTGCGCTCGCGTCGGGCAGCGTATTAGCACGCACGACAGCGGGTCCACTACCTGCACGGCCGAGGACATCCTCACCGACGAGAAGCGCGACATCCTGGTCAAGCACCTCATCCCGCAGGCGCTGCAGCTGCACACGGAGCGGCTGAAGGTGCGGCAGGTGCAGGACAAGTGGAAGGTGACGGGCATGGACGACGATGTGTGCAGCGACTTCAAGGTGCCGCCGGCGCACATCACCGATGGCCTGAGCAACACCGACTTCGTGATGTACGTCGCCTCCGTGCCGAGCGAGGAGGGTGTGCTGGCGTGGGCCGCGACCTGCCAGGTGTTCTCTGACGGCCATCCAGCCGTGGGCGTCGTCAACATCCCCGCGGCGAACATTGCGTCGCGGTACAACCAGCTGGTGACGCGTGTCGTCACGCACGAGATGGCGCACACGCTCGGCTTCAGCGTCGACTTCTTCCAAGACGCCAGCATCATGCACCAGGTGTCGAACATTCGGCGCAAGACTTCAAAAGTACCTGTGTTGAAAAGCCGCACGGCGGTGGCGAAGGCGCGCGAGCAGTACGGCTGCGACACCTTGGAGTATCTGGAGATCGAGGACCAGGGCGGTGCGGGCTCCGCCGGGTCGCACATCAAGATGCGCAACGCGCAGGACGAGCTCATGGCGCCTGCCGCAGCTGCCGGGTACTACAGCGCCCTGACCATGGCCATCTTCCAGGACCTCGGCTTCTACCAGGCGGACTTCAGCAAGGCCGAGGTGATGCCGTGGGGCCGGAACGCCGGCTGCGCCTTCCTCAGCGAGAAGTGCATGGAGCGGAACATCACGAAGTGGCCGGCGATGTTCTGCAATGAGAACGAGGTGACTATGCGCTGCCCCACCAGTCGTCTCAGCCTTGGAAAGTGCGGTGTTACCCGTCACCCGGACCTTCCGCCGTACTGGCAGTACTTCACGGACCCGTCCCTCGCCGGCATCTCCGCCTTCATGGACTGCTGCCCTGTCGTGGAGCCCTACGGTGATGGCAGCTGCGCACAGCGTGCGTCTGAAGCGGGCGCACCATTCAAAGGCTTCAACGTCTTCTCCGACGCGGCGCGCTGCATCGATGGCGCCTTCAGGCCGAAGACGAGTCACGGCATAATCAAGTCGTACGCCGGACTGTGCGCCAACGTGCGGTGCGACACGGCCACGCGCACGTACAGCGTGCAGGTGCACGGCGGCAGCGGCTACGCCAACTGCACGCCGGGCCTCAGAGTTGAGCTGAGCACCGTGAGCAGCGCCTTCGAGGAGGGCGGCTACATCACGTGCCCGCCGTACGTGGAGGTGTGCCAGGGCAACGTGCAGGCTGCCAAGGACGGCGGCAACGCCGCGGCTGGTCGCCGTGGTCCGCGCGCCGCGGCGACGGCGCTGCTGGTGGCCGCGCTGCTGGCCGTGGCGCTCTAG

> Variant_2

ATGTCCGTCGACAGCAGCAGCACGCACCGGCACCGcAGCGTCGCCGCGCGCCTGGTGCGCCTCGCGGCTGCCGGCGCCGCAGTCATCGCTGCTGTCGGCACCGCGGCCGCGTGGGCACACGCCGGTGCGGTGCAGCACCGCTGCATCCACGACGCGATGCAGGCACGCGTGCGGCAGTCGGTGGCGCGCCACCACACGGCCCCCGGCGCCGTGTCCGCGGTGGGCCTGCCGTACGTTACTCTCGACACCGCGGCCGCCGCCGATCGCCGGCCGGGCAGCGCGCCTACAGTCGTGCGCGCCGCGAACTGGGGCGCGCTGCGCATCGCCGTCTCCACCGAGGACCTCACCGACCCCGCCTACCACTGCGCTCGCGTCGGGCAGCGTATTAGCACGCACGACAGCGGGTCCACTACCTGCACGGCCGAGGACATCCTCACCGACGAGAAGCGCGACATCCTGGTCAAGCACCTCATCCCGCAGGCGCTGCAGCTGCACACGGAGCGGCTGAAGGTGCGGCAGGTGCAGGACAAGTGGAAGGTGACGGGCATGGACGACGATGTGTGCAGCGACTTCAAGGTGCCGCCGGCGCACATCACCGATGGCCTGAGCAACACCGACTTCGTGATGTACGTCGCCTCCGTGCCGAGCGAGGAGGGTGTGCTGGCGTGGGCCGCGACCTGCCAGGTGTTCTCTGACGGCCATCCAGCCGTGGGCGTCGTCAACATCCCCGCGGCGAACATTGCGTCGCGGTACAACCAGCTGGTGACGCGTGTCGTCACGCACGAGATGGCGCACACGCTCGGCTTCAGCGTCGACTTCTTCCAAGACGCCAGCATCATGCACCAGGTGTCGAACATTCGGCGCAAGACTTCAAAAGTACCTGTGTTGAAAAGCCGCACGGCGGTGGCGAAGGCGCGCGAGCAGTACGGCTGCGACACCTTGGAGTATCTGGAGATCGAGGACCAGGGCGGTGCGGGCTCCGCCGGGTCGCACATCAAGATGCGCAACGCGCAGGACGAGCTCATGGCGCCTGCCGCAGCTGCCGGGTACTACAGCGCCCTGACCATGGCCATCTTCCAGGACCTCGGCTTCTACCAGGCGAACTTCAGCAAGGCCGAGGTGATGCCGTGGGGCCGGAACGCCGGCTGCGCCTTCCTCAGCGAGAAGTGCATGGAGCGGAACATCACGAAGTGGCCGGCGATGTTCTGCAATGAGAACGAGGTGGCTATGCGCTGCCCCACCAGTCGTCTCAGCCTTGGAAAGTGCGGTGTTACCCGTCACCCGGACCTTCCGCCGTACTGGCAGTACTTCACGGACCCGTCCCTCGCCGGCATCTCCGCCTTCATGGACTGCTGCCCTGTCGTGGAGCCCTACGGTGATGGCAGCTGCGCACAGCGTGCGTCTGAAGCGGGCGCACCATTCAAAGGCTTCAACGTCTTCTCCGACGCGGCGCGCTGCATCGATGGCGCCTTCAGGCCGAAGACGAGTCACGGCATAATCAAGTCGTACGCCGGACTGTGCGCCAACGTGCGGTGCGACACGGCCACGCGCACGTACAGCGTGCAGGTGCACGGCGGCAGCGGCTACGCCAACTGCACGCCGGGCCTCAGAGTTGAGCTGAGCACCGTGAGCAGCGCCTTCGAGGAGGGCGGCTACATCACGTGCCCGCCGTACGTGGAGGTGTGCCAGGGCAACGTGCAGGCTGCCAAGGACGGCGGCAACGCCGCGGCTGGTCGCCGTGGTCCGCGCGCCGCGGCGACGGCGCTGCTGGTGGCCGCGCTGCTGGCCGTGGCGCTCTAG

> Variant_3

ATGTCCGTCGACAGCAGCAGCACGCACCGGCACCGCAGCGTCGCCGCGCGCCTGGTGCGCCTCGCGGCTGCCGGCGCCGCAGTCATCGCTGCTGTCGGCACCGCGGCCGCGTGGGCACACGCCGGTGCGGTGCAGCACCGCTGCATCCACGACGCGATGCAGGCACGCGTGCGGCAGTCGGTGGCGCGCCACCACACGGCCCCCGGCGCCGTGTCCGCGGTGGGCCTGCCGTACGTTACTCTCGACACCGCGGCCGCCGCCGATCGCCGGCCGGGCAGCGCGCCTACAGTCGTGCGCGCCGCGAACTGGGGCGCGCTGCGCATCGCCGTCTCCACCGAGGACCTCACCGACCCCGCCTACCACTGCGCTCGCGTCGGGCAGCGTATTAGCACGCACGACAGCGGGTCCACTACCTGCACGGCCGAGGACATCCTCACCGACGAGAAGCGCGACATCCTGGTCAAGCACCTCATCCCGCAGGCGCTGCAGCTGCACACGGAGCGGCTGAAGGTGCGGCAGGTGCAGGACAAGTGGAAGGTGACGGGCATGGACGACGATGTGTGCAGCGACTTCAAGGTGCCGCCGGCGCACATCACCGATGGCCTGAGCAACACCGACTTCGTGATGTACGTCGCCTCCGTGCCGAGCGAGGAGGGTGTGCTGGCGTGGGCCGCGACCTGCCAGGTGTTCTCTGACGGCCATCCAGCCGTGGGCGTCGTCAACATCCCCGCGGCGAACATTGCGTCGCGGTACAACCAGCTGGTGACGCGTGTCGTCACGCACGAGATGGCGCACACGCTCGGCTTCAGCGTCGACTTCTTCCAAGACGCCAGCATCATGCACCAGGTGTCGAACATTCGGCGCAAGACTTCAAAAGTACCTGTGTTGAAAAGCCGCACGGCGGTGGCGAAGGCGCGCGAGCAGTACGGCTGCGACACCTTGGAGTATCTGGAGATCGAGGACCAGGGCGGTGCGGGCTCCGCCGGGTCGCACATCAAGATGCGCAACGCGCAGGACGAGCTCATGGCGCCTGCCGCAGCTGCCGGGTACTACAGCGCCCTGACCATGGCCATCTTCCAGGACCTCGGCTTCTACCAGGCGGACTTCAGCAAGGCCGAGGTGATGCCGTGGGGCCGGAACGCCGGCTGCGCCTTCCTCAGCGAGAAGTGCATGGAGCGGAACATCACGAAGTGGCCGGCGATGTTCTGCAATGAGAACGAGGTGGCTATGCGCTGCCCCACCAGTCGTCTCAGCCTTGGAAAGTGCGGTGTTACCCGTCACCCGGACCTTCCGCCGTACTGGCAGTACTTCACGGACCCGTCCCTCGCCGGCATCTCCGCCTTCATGGACTGCTGCCCTGTCGTGGAGCCCTACGGTGATGGCAGCTGCGCACAGCGTGCGTCTGAAGCGGGCGCACCATTCAAAGGCTTCAACGTCTTCTCCGACGCGGCGCGCTGCATCGATGGCGCCTTCAGGCCGAAGACGAGTCACGGCATAATCAAGTCGTACGCCGGACTGTGCGCCAACGTGCGGTGCGACACGGCCACGCGCACGTACAGCGTGCAGGTGCACGGCGGCAGCGGCTACGCCAACTGCACGCCGGGCCTCAGAGTTGAGCTGAGCACCGTGAGCAGCGCCTTCGAGGAGGGCGGCTACATCACGTGCCCGCCGTACGTGGAGGTGTGCCAGGGCAACGTGCAGGCTGCCAAGGACGGCGGCAACGCCGCGGCTGGTCGCCGTGGTCCGCGCGCCGCGGCGACGGCGCTGCTGGTGGCCGCGCTGCTGGCCGTGGCGCTCTAG

> Variant_4

ATGTCCGTCGACAGCAGCAGCACGCACCGGCACCGCAGCGTCGCCGCGCGCCTGGTGCGCCTCGCGGCTGCCGGCGCCGCAGTCATCGCTGCTGTCGGCACCGCGGCCGCGTGGGCACACGCCGGTGCGGTGCAGCACCGCTGCATCCACGACGCGATGCAGGCACGCGTGCGGCAGTCGGTGGCGCGCCACCACACGGCCCCCGGCGCCGTGTCCGCGGTGGGCCTGCCGTACGTTACTCTCGACACCGCGGCCGCCGCCGATCGCCGGCCGGGCAGCGCGCCTACAGTCGTGCGCGCCGCGAACTGGGGCGCGCTGCGCATCGCCGTCTCCACCGAGGACCTCACCGACCCCGCCTACCACTGCGCTCGCGTCGGGCAGCGTATTAGCACGCACGACAGCGGGTCCACTACCTGCACGGCCGAGGACATCCTCACCGACGAGAAGCGCGACATCCTGGTCAAGCACCTCATCCCGCAGGCGCTGCAGCTGCACACGGAGCGGCTGAAGGTGCGGCAGGTGCAGGACAAGTGGAAGGTGACGGGCATGGACGACGATGTGTGCAGCGACTTCAAGGTGCCGCCGGCGCACATCACCGATGGCCTGAGCAACACCGACTTCGTGATGTACGTCGCCTCCGTGCCGAGCGAGGAGGGTGTGCTGGCGTGGGCCGCGACCTGCCAGGTGTTCTCTGACGGCCATCCAGCCGTGGGCGTCGTCAACATCCCCGCGGCGAACATTGCGTCGCGGTACAACCAGCTGGTGACGCGTGTCGTCACGCACGAGATGGCGCACACGCTCGGCTTCAGCGTCGACTTCTTCCAAGACGCCAGCATCATGCACCAGGTGTCGAACATTCGGCGCAAGACTTCAAAAGTACCTGTGTTGAAAAGCCGCACGGCGGTGGCGAAGGCGCGCGAGCAGTACGGCTGCGACACCTTGGAGTATCTGGAGATCGAGGACCAGGGCGGTGCGGGCTCCGCCGGGTCGCACATCAAGATGCGCAACGCGCAGGACGAGCTCATGGCGCCTGCCGCAGCTGCCGGGTACTACAGCGCCCTGACCATGGCCATCTTCCAGGACCTCGGCTTCTACCAGGCGGACTTCAGCAAGGCCGAGGTGATGCCGTGGGGCCGGAACGCCGGCTGCGCCTTCCTCAGCGAGAAGGGCATGGAGCGGAACATCCCGAAGTGGCCGGCGATGTTCTGCAATGAGAACGAGGTGGCTATGCGCTGCCCCACCAGTCGTCTCAGCCTTGGAAAGTGCGGTGTTACCCGTCACCCGGACCTTCCGCCGTACTGGCAGTACTTCACGGACCCGTCCCTCGCCGGCATCTCCGCCTTCATGGACTGCTGCCCTGTCGTGGAGCCCTACGGTGATGGCAGCTGCGCACAGCGTGCGTCTGAAGCGGGCGCACCATTCAAAGGCTTCAACGTCTTCTCCGACGCGGCGCGCTGCATCGATGGCGCCTTCAGGCCGAAGACGAGTCACGGCATAATCAAGTCGTACGCCGGACTGTGCGCCAACGTGCGGTGCGACACGGCCACGCGCACGTACAGCGTGCAGGTGCACGGCGGCAGCGGCTACGCCAACTGCACGCCGGGCCTCAGAGTTGAGCTGAGCACCGTGAGCAGCGCCTTCGAGGAGGGCGGCTACATCACGTGCCCGCCGTACGTGGAGGTGTGCCAGGGCAACGTGCAGGCTGCCAAGGACGGCGGCAACGCCGCGGCTGGTCGCCGTGGTCCGCGCGCCGCGGCGACGGCGCTGCTGGTGGCCGCGCTGCTGGCCGTGGCGCTCTAG
