# Supplementary material for: In silico identification of Leishmania GP63 protein epitopes to generate a new vaccine antigen against leishmaniasis
Source: PLoS Negl Trop Dis. 2025 Jun 5;19(6):e0013137. doi: 10.1371/journal.pntd.0013137 (PMC12140206; doi:10.1371/journal.pntd.0013137)
Supplement: S3 File — (DOCX) [file pntd.0013137.s003.docx]

L.major

>Variant_1

ATGTCCGTGGACAGCAGCAGCACGCACCGGCGCCGCTGCGTCGCCGCGCGCCTGGTGCGCCTCGCGGCTGCCGGCGCCGCAGTCACCGTTGCTGTCGGCACCGCGGCCGCGTGGGCACACGCCGGTGCGCTGCAGCACCGCTGCGTCCACGACGCGATGCAGGCACGCGTGCGGCAGTCGGTGGCGGACCACCACAAGGCCCCCGGCGCGGTGTCCGCGGTGGGTCTGCCGTACGTTACTCTCGACGCCGCGCACACCGCGGCCGCCGCCGATCCCAGGCCGGGCAGCGCGCGCAGCGTCGTGCGCGACGTGAACTGGGGCGCGCTGCGCATCGCCGTCTCCACCGAGGACCTCACCGACCCCGCCTACCACTGCGCTCGCGTCGGGCAGCATGTCAAAGACCACGCCGGCGCCATCGTCACCTGCACCGCCGAGGACATCCTCACCAACGAGAAGCGCGACATCCTGGTCAAGCACCTCATCCCGCAGGCGGTGCAGCTGCACACGGAGCGGCTGAAGGTGCAGCAGGTGCAGGGCAAGTGGAAGGTGACGGACATGGTCGGCGAGATCTGTGGCGACTTCAAGGTGCCGCAGGCGCACATCACCGAGGGCTTCAGCAACACCGACTTCGTGATGTACGTCGCCTCCGTGCCGAGTGAGGAGGGTGTGCTGGCGTGGGCCACGACCTGCCAGACGTTCTCTGACGGCCATCCAGCCGTGGGCGTCATCAACATCCCCGCGGCGAACATTGCGTCGCGGTACGACCAGCTCGTCACGCGTGTCGTCACGCACGAGATGGCGCACGCGCTCGGCTTCAGCGGCCCATTCTTCGAGGACGCCCGCATCGTGGCGAGCGTTCCGAACGTTCGAGGCAAGAACTTCGATGTTCCCGTGATCAACAGCAGCACGGCAGTGGCGAAGGCGCGCGAGCAGTACGGCTGCGACACTTTGGAGTATCTGGAGATGGAGGACCAAGGCAGTGCGGGCTCCGCCGGGTCGCACATCAAGATGCGCAACGCGCAGGACGAGCTCATGGCGGCAGCCAGTGGTGCCGGGTACTACACCGCCCTGACCATGGCCATCCTCCAGGACCTCGGCTTCTACCAGGCGGACTTCAGCAAGGCCGAGGTGATGCCGTGGGGCCAGAACGCCGGCTGCGCCTTCCTCACCAACAAGTGCATGGAGCAGAACATCACGCAGTGGCCGGCGATGTTCTGCAATGAGAGCGAGGACGCCATCCGCTGCCCCACCAGTCGTCTCAGCCTCGGTGCATGCGGTGTTACCCGTCACCCGGGCCTTCCGCCGTACTGGCAGTACTTCACGGACCCGTCCCTCGCCGGCGTCTCCGCCTTCATGGACTACTGCCCTGTCGTGGTGCCCTACAGTGATATCAGCTGCACGCAGCGTGCCTCTGAGGCACATGCTTCGTTGCTGCCCTTCAACGTCTTCTCTGACGCGGCGCGCTGCATCGATGGTGCCTTCAGACCGAAGGCAACTAACGGCATAGTCAAGTCGTACGCCGGCCTGTGCGCCAACGTGCAGTGTGACACGGCCACACGCACGTACAGCGTGCAGGTGCACGGCAGTAACGACTACACCAACTGCACGCCGGGCCTCAGAGTTGAGCTGAGCACCGTGAGCAACGCCTTCGAGGGGGGCGGCTACATCACGTGCCCGCCGTACGTGGAGGTGTGCCAGGGCAACGTGCAGGCTGCCAAGGACGGCGGCAACACGGCGGCTGGTCGTCGTGGTCCGCGCGCCGCGGCGACGGCGCTGCTGGTGGCCGCGCTGCTGGCCGTGGCGCTCTAG

>Variant_2

ATGTCCGTGGACAGCAGCAGCACGCACCGGCGCCGCTGCGTCGCCGCGCGCCTGGTGCGCCTCGCGGCTGCCGGCGCCGCAGTCACCGTTGCTGTCGGCACCGCGGCCGCGTGGGCACACGCCGGTGCGCTGCAGCACCGCTGCGTCCACGACGCGATGCAGGCACGCGTGCGGCAGTCGGTGGCGGACCACCACAAGGCCCCCGGCGCGGTGTCCGCGGTGGGTCTGCCGTACGTTACTCTCGACGCCGCGCACACCGCGGCCGCCGCCGATCCCAGGCCGGGCAGCGCGCGCAGCGTCGTGCGCGACGTGAACTGGGGCGCGCTGCGCATCGCCGTCTCCACCGAGGACCTCACCGACCCCGCCTACCACTGCGCTCGCGTCGGGCAGCATGTCAAAGACCACGCCGGCGCCATCGTCACCTGCACCGCCGAGGACATCCTCACCAACGAGAAGCGCGACATCCTGGTCAAGCACCTCATCCCGCAGGCGGTGCAGCTGCACAAGGAGCGGCTGAAGGTGCAGCAGGTGCAGGGCAAGTGGAAGGTGACGGACATGGTCGGCGAGATCTGTGGCGACTTCAAGGTGCCGCAGGCGCACATCACCGAGGGCTTCGGCAACACCGACTTCGTGATGTACGTCGCCTCCGTGCCGAGTGAGGAGGGTGTGCTGGCGTGGGCCACGACCTGCCAGACGTTCTCTGACGGCCATCCAGCCGTGGGCGTCATCAACATCCCCGCGGCGAACATTGCGTCGCGGTACGACCAGCTCGTCACGCGTGTCGTCACGCACGAGATGGCGCACGCGCTCGGCTTCAGCGGCCTATTCTTCAAAGACGCCCGCATCGTGGCGAACGTTCCGAACGTTCGAGGCAAGAACTTCGATGTTCCCGTGATCAACAGCAGCACGGCAGTGGCGAAGGCGCGCGAGCAGTACGGCTGCGACACTTTGGAGTATCTGGAGATGGAGGACCAAGGCAGTGCGGGCTCCGCCGGGTCGCACATCAAGATGCGCAACGCGCAGGACGAGCTCATGGCGGCAGCCAGTGGTGCCGGGTACTACACTGCCCTGACCATGGCCATCTTCCAGGACCTCGGCTTCTACCAGGCGGACTTCAGCAAGGCCGAGGTGATGCCGTGGGGCCAGAACGCCGGCTGCGCCTTCCTCACCAACAAGTGCATGGAGCAGAGCGTCACGCAGTGGCCGGCGATGTTCTGCAATGAGAGCGAGGACGCCATCCGCTGCCCCACCAGTCGTCTCAGCCTCGGTGCATGCGGTGTTACCCGTCACCCGGGCCTTCCGCCGTACTGGCAGTACTTCACGGACCCGTCCCTCGCCGGCCTCTCCGCCTTCATGGACTACTGCCCTGTCGTGGTGCCCTACAGTGATATCAGCTGCACGCAGCGTGCCTCTGAGGCACATGCTTCGTTGCTGCCCTTCAACGTCTTCTCTGACGCGGCGCGCTGCATCGATGGTGCCTTCAGACCGAAGGCAACTAACGGCATAGTCAAGTCGTACGCCGGCCTGTGCGCCAACGTGCAGTGTGACACGGCCACACGCACGTACAGCGTGCAGGTGCACGGCAGTAACGACTACACCAACTGCACGCCGGGCCTCAGAGTTGAGCTGAGCACCGTGAGCAACGCCTTCGAGGGGGGCGGCTACATCACGTGCCCGCCGTACGTGGAGGTGTGCCAGGGCAACGTGCAGGCTGCCAAGGACGGCGGCAACACGGCGGCTGGTCGTCGTGGTCCGCGCGCCGCGGCGACGGCGCTGCTGGTGGCCGCGCTGCTGGCCGTGGCGCTCTAG

>Variant_3

ATGTCCGTGGACAGCAGCAGCACGCACCGGCGCCGCTGCGTCGCCGCGCGCCTGGTGCGCCTCGCGGCTGCCGGCGCCGCAGTCACCGTTGCTGTCGGCACCGCGGCCGCGTGGGCACACGCCGGTGCGCTGCAGCACCGCTGCGTCCACGACGCGATGCAGGCACGCGTGCGGCAGTCGGTGGCGGACCACCACAAGGCCCCCGGCGCGGTGTCCGCGGTGGGTCTGCCGTACGTTACTCTCGACGCCGCGCACACCGCGGCCGCCGCCGATCCCAGGCCGGGCAGCGCGCGCAGCGTCGTGCGCGACGTGAACTGGGGCGCGCTGCGCATCGCCGTCTCCACCGAGGACCTCACCGACCCCGCCTACCACTGCGCTCGCGTCGGGCAGCATGTCAAAGACCACGCCGGCGCCATCGTCACCTGCACCGCCGAGGACATCCTCACCAACGAGAAGCGCGACATCCTGGTCAAGCACCTCATCCCGCAGGCGGTGCAGCTGCACAAGGAGCGGCTGAAGGTGCAGCAGGTGCAGGGCAAGTGGAAGGTGACGGACATGGTCGGCGAGATCTGTGGCGACTTCAAGGTGCCGCAGGCGCACATCACCGAGGGCTTCGGCAACACCGACTTCGTGATGTACGTCGCCTCCGTGCCGAGTGAGGAGGGTGTGCTGGCGTGGGCCACGACCTGCCAGACGTTCTCTGACGGCCATCCAGCCGTGGGCGTCATCAACATCCCCGCGGCGAACATTGCGTCGCGGTACGACCAGCTCGTCACGCGTGTCGTCACGCACGAGATGGCGCACGCGCTCGGCTTCAGCGGCCTATTCTTCAAAGACGCCCGCATCGTGGCGAACGTTCCGAACGTTCGAGGCAAGAACTTCGATGTTCCCGTGATCAACAGCAGCACGGCAGTGGCGAAGGCGCGCGAGCAGTACGGCTGCGACACTTTGGAGTATCTGGAGATGGAGGACCAAGGCAGTGCGGGCTCCGCCGGGTCGCACATCAAGATGCGCAACGCGCAGGACGAGCTCATGGCGGCAGCCAGTGGTGCCGGGTACTACACTGCCCTGACCATGGCCATCTTCCAGGACCTCGGCTTCTACCAGGCGGACTTCAGCAAGGCCGAGGTGATGCCGTGGGGCCAGAACGCCGGCTGCGCCTTCCTCACCAACAAGTGCATGGAGCAGAACGTCACGCAATGGCCGGCGATGTTCTGCAATGAGAGCGAGGACGCCATCCGCTGCCCCACCAGTCGTCTCAGCCTCGGTGCATGCGGTGTTACCCGTCACCCGGGCCTTCCGCCGTACTGGCAGTACTTCACGGACCCGTCCCTCGCCGGCCTCTCCGCCTTCATGGACTACTGCCCTGTCGTGGTGCCCTACAGTGATATCAGCTGCACGCAGCGTGCCTCTGAGGCACATGCTTCGTTGCTGCCCTTCAACGTCTTCTCTGACGCGGCGCGCTGCATCGATGGTGCCTTCAGACCGAAGGCAACTAACGGCATAGTCAAGTCGTACGCCGGCCTGTGCGCCAACGTGCAGTGTGACACGGCCACACGCACGTACAGCGTGCAGGTGCACGGCAGTAACGACTACACCAACTGCACGCCGGGCCTCAGAGTTGAGCTGAGCACCGTGAGCAACGCCTTCGAGGGGGGCGGCTACATCACGTGCCCGCCGTACGTGGAGGTGTGCCAGGGCAACGTGCAGGCTGCCAAGGACGGCGGCAACACGGCGGCTGGTCGTCGTGGTCCGCGCGCCGCGGCGACGGCGCTGCTGGTGGCCGCGCTGCTGGCCGTGGCGCTCTAG

>Variant_4

ATGTCCGTGGACAGCAGCAGCACGCACCGGCGCCGCTGCGTCGCCGCGCGCCTGGTGCGCCTCGCGGCTGCCGGCGCCGCAGTCACCGTTGCTGTCGGCACCGCGGCCGCGTGGGCACACGCCGGTGCGCTGCAGCACCGCTGCGTCCACGACGCGATGCAGGCACGCGTGCGGCAGTCGGTGGCGGACCACCACAAGGCCCCCGGCGCGGTGTCCGCGGTGGGTCTGCCGTACGTTACTCTCGACGCCGCGCACACCGCGGCCGCCGCCGATCCCAGGCCGGGCAGCGCGCGCAGCGTCGTGCGCGACGTGAACTGGGGCGCGCTGCGCATCGCCGTCTCCACCGAGGACCTCACCGACCCCGCCTACCACTGCGCTCGCGTCGGGCAGCATGTCAAAGACCACGCCGGCGCCATCGTCACCTGCACCGCCGAGGACATCCTCACCAACGAGAAGCGCGACATCCTGGTCAAGCACCTCATCCCGCAGGCGGTGCAGCTGCACAAGGAGCGGCTGAAGGTGCAGCAGGTGCAGGGCAAGTGGAAGGTGACGGACATGGTCGGCGAGATCTGTGGCGACTTCAAGGTGCCGCAGGCGCACATCACCGAGGGCTTCGGCAACACCGACTTCGTGATGTACGTCGCCTCCGTGCCGAGTGAGGAGGGTGTGCTGGCGTGGGCCACGACCTGCCAGACGTTCTCTGACGGCCATCCAGCCGTGGGCGTCATCAACATCCCCGCGGCGAACATTGCGTCGCGGTACGACCAGCTCGTCACGCGTGTCGTCACGCACGAGATGGCGCACGCGCTCGGCTTCAGCGGCCCATTCTTCGAGGACGCCCGCATCGTGGCGAACGTTTCGAACGTTCGAGGCAAGAACTTCGATGTTCCCGTGATCAACAGCAGCACGGCAGTGGCGAAGGCGCGCGAGCAGTACGGCTGCGACACTTTGGAGTATCTGGAGATGGAGGACCAAGGCAGTGCGGGCTCCGCCGGGTCGCACATCAAGATGCGCAACGCGCAGGACGAGCTCATGGCGGCAGCCAGTGGTGCCGGGTACTACACCGCCCTGACCATGGCCATCTTCCAGGACCTCGGCTTCTACCAGGCGGACTTCAGCAAGGCCGAGGTGATGCCGTGGGGCCAGAACGCCGGCTGCGCCTTCCTCACCAACAAGTGCATGGAGCAGAGCGTCACGCAGTGGCCGGCGATGTTCTGCAATGAGAGCGAGGACGCCATCCGCTGCCCCACCAGTCGTCTCAGCCTCGGTGCATGCGGTGTTACCCGTCACCCGGGCCTTCCGCCGTACTGGCAGTACTTCACGGACCCGTCCCTCGCCGGCGTCTCCGCCTTCATGGACTACTGCCCTGTCGTGGTGCCCTACAGTGATATCAGCTGCACGCAGCGTGCCTCTGAGGCACATGCTTCGTTGCTGCCCTTCAACGTCTTCTCTGACGCGGCGCGCTGCATCGATGGTGCCTTCAGACCGAAGGCAACTGACGGCATAGTCAAGTCGTACGCCGGCCTGTGCGCCAACGTGCAGTGTGACACGGCCACACGCACGTACAGCGTGCAGGTGCACGGCAGTAACGACTACACCAACTGCACGCCGGGCCTCAGAGTTGAGCTGAGCACCGTGAGCAACGCCTTCGAGGGGGGCGGCTACATCACGTGCCCGCCGTACGTGGAGGTGTGCCAGGGCAACGTGCAGGCTGCCAAGGACGGCGGCAACACGGCGGCTGGTCGTCGTGGTCCGCGCGCCGCGGCGACGGCGCTGCTGGTGGCCGCGCTGCTGGCCGTGGCGCTCTAG

>Variant_5

ATGTCCGTGGACAGCAGCAGCACGCACCGGCGCCGCTGCGTCGCCGCGCGCCTGGTGCGCCTCGCGGCTGCCGGCGCCGCAGTCACCGTTGCTGTCGGCACCGCGGCCGCGTGGGCACACGCCGGTGCGCTGCAGCACCGCTGCGTCCACGACGCGATGCAGGCACGCGTGCGGCAGTCGGTGGCGGACCACCACAAGGCCCCCGGCGCGGTGTCCGCGGTGGGTCTGCCGTACGTTACTCTCGACGCCGCGCACACCGCGGCCGCCGCCGATCCCAGGCCGGGCAGCGCGCGCAGCGTCGTGCGCGACGTGAACTGGGGCGCGCTGCGCATCGCCGTCTCCACCGAGGACCTCACCGACCCCGCCTACCACTGCGCTCGCGTCGGGCAGCATGTCAAAGACCACGCCGGCGCCATCGTCACCTGCACCGCCGAGGACATCCTCACCAACGAGAAGCGCGACATCCTGGTCAAGCACCTCATCCCGCAGGCGGTGCAGCTGCACACGGAGCGGCTGAAGGTGCAGCAGGTGCAGGGCAAGTGGAAGGTGACGGACATGGTCGGCGAGATCTGTGGCGACTTCAAGGTGCCGCAGGCGCACATCACCGAGGGCTTCAGCAACACCGACTTCGTGATGTACGTCGCCTCCGTGCCCAATGAAGAGGGTGTGCTGGCGTGGGCCACGACCTGCCAGACGTTCTCTGACGGCCATCCAGCCGTGGGCGTCATCAACATCCCCGCGGCGAACATTGCGTCGCGGTACGACCAGCTCGTCACGCGTGTCGTCACGCACGAGATGGCGCACGCGCTCGGCTTCAGCGGCCTATTCTTCAGAGACGCCCGCATCGTGGCGAACGTTCCGAACGTTCGAGGCAAGAACTTCGATGTTCCCGTGATCAACAGCAGCACGGCAGTGGCGAAGGCGCGCGAGCAGTACGGCTGCGACACTTTGGAGTATCTGGAGATGGAGGACCAAGGCAGTGCGGGCTCCGCCGGGTCGCACATCAAGATGCGCAACGCGCAGGACGAGCTCATGGCGGCAGCCAGTGGTGCCGGGTACTACACTGCCCTGACCATGGCCATCTTCCAGGACCTCGGCTTCTACCAGGCGGACTTCAGCAAGGCCGAGGTGATGCCGTGGGGCCAGAACGCCGGCTGCGCCTTCCTCACCAACAAGTGCATGGAGCAGAACATCACGCAGTGGCCGGCGATGTTCTGCAATGAGAGCGAGGACGCCATCCGCTGCCCCACCAGTCGTCTCAGCCTCGGTGCATGCGGTGTTACCCGTCACCCGGGCCTTCCGCCGTACTGGCAGTACTTCACGGACCCGTCCCTCGCCGGCGTCTCCGCCTTCATGGACTACTGCCCTGTCGTGGTGCCCTACAGTGATATCAGCTGCACGCAGCGTGCCTCTGAGGCACATGCTTCGTTGCTGCCCTTCAACGTCTTCTCTGACGCGGCGCGCTGCATCGATGGTGCCTTCAGACCGAAGGCAACTAACGGCATAGTCAAGTCGTACGCCGGCCTGTGCGCCAACGTGCAGTGTGACACGGCCACACGCACGTACAGCGTGCAGGTGCACGGCAGTAACGACTACACCAACTGCACGCCGGGCCTCAGAGTTGAGCTGAGCACCGTGAGCAACGCCTTCGAGGGGGGCGGCTACATCACGTGCCCGCCGTACGTGGAGGTGTGCCAGGGCAACGTGCAGGCTGCCAAGGACGGCGGCAACACGGCGGCTGGTCGTCGTGGTCCGCGCGCCGCGGCGACGGCGCTGCTGGTGGCCGCGCTGCTGGCCGTGGCGCTCTAG

>Variant_6

ATGTCCGTGGACAGCAGCAGCACGCACCGGCGCCGCTGCGTCGCCGCGCGCCTGGTGCGCCTCGCGGCTGCCGGCGCCGCAGTCACCGTTGCTGTCGGCACCGCGGCCGCGTGGGCACACGCCGGTGCGCTGCAGCACCGCTGCGTCCACGACGCGATGCAGGCACGCGTGCGGCAGTCGGTGGCGGACCACCACAAGGCCCCCGGCGCGGTGTCCGCGGTGGGTCTGCCGTACGTTACTCTCGACGCCGCGCACACCGCGGCCGCCGCCGATCCCAGGCCGGGCAGCGCGCGCAGCGTCGTGCGCGACGTGAACTGGGGCGCGCTGCGCATCGCCGTCTCCACCGAGGACCTCACCGACCCCGCCTACCACTGCGCTCGCGTCGGGCAGCATGTCAAAGACCACGCCGGCGCCATCGTCACCTGCACCGCCGAGGACATCCTCACCAACGAGAAGCGCGACATCCTGGTCAAGCACCTCATCCCGCAGGCGGTGCAGCTGCACAAGGAGCGGCTGAAGGTGCAGCAGGTGCAGGGCAAGTGGAAGGTGACGGACATGGTCGGCGAGATCTGTGGCGACTTCAAGGTGCCGCAGGCGCACATCACCGAGGGCTTCAGCAACACCGACTTCGTGATGTACGTCGCCTCCGTGCCGAGTGAGGAGGGTGTGCTGGCGTGGGCCACGACCTGCCAGACGTTCTCTGACGGCCATCCAGCCGTGGGCGTCATCAACATCCCCGCGGCGAACATTGCGTCGCGGTACGACCAGCTCGTCACGCGTGTCGTCACGCACGAGATGGCGCACGCGCTCGGCTTCAGCGGCCTATTCTTCAAAGACGCCCGCATCGTGGCGAACGTTCCGAACGTTCGAGGCAAGAACTTCGATGTTCCCGTGATCAACAGCAGCACGGCAGTGGCGAAGGCGCGCGAGCAGTACGGCTGCGACACTTTGGAGTATCTGGAGATGGAGGACCAAGGCAGTGCGGGCTCCGCCGGGTCGCACATCAAGATGCGCAACGCGCAGGACGAGCTCATGGCGGCAGCCAGTGGTGCCGGGTACTACACCGCCCTGACCATGGCCATCCTCCAGGACCTCGGCTTCTACCAGGCGGACTTCAGCAAGGCCGAGGTGATGCCGTGGGGCCAGAACGCCGGCTGCGCCTTCCTCACCAACAAGTGCATGGAGCAGAGCGTCACGCAGTGGCCGGCGATGTTCTGCAATGAGAGCGAGGACGCCATCCGCTGCCCCACCAGTCGTCTCAGCCTCGGTGCATGCGGTGTTACCCGTCACCCGGGCCTTCCGCCGTACTGGCAGTACTTCACGGACCCGTCCCTCGCCGGCCTCTCCGCCTTCATGGACTACTGCCCTGTCGTGGTGCCCTACAGTGATATCAGCTGCACGCAGCGTGCCTCTGAGGCACATGCTTCGTTGCTGCCCTTCAACGTCTTCTCTGACGCGGCGCGCTGCATCGATGGTGCCTTCAGACCGAAGGCAACTAACGGCATAGTCAAGTCGTACGCCGGCCTGTGCGCCAACGTGCAGTGTGACACGGCCACACGCACGTACAGCGTGCAGGTGCACGGCAGTAACGACTACACCAACTGCACGCCGGGCCTCAGAGTTGAGCTGAGCACCGTGAGCAACGCCTTCGAGGGGGGCGGCTACATCACGTGCCCGCCGTACGTGGAGGTGTGCCAGGGCAACGTGCAGGCTGCCAAGGACGGCGGCAACACGGCGGCTGGTCGTCGTGGTCCGCGCGCCGCGGCGACGGCGCTGCTGGTGGCCGCGCTGCTGGCCGTGGCGCTCTAG
