# Supplementary material for: In silico identification of Leishmania GP63 protein epitopes to generate a new vaccine antigen against leishmaniasis
Source: PLoS Negl Trop Dis. 2025 Jun 5;19(6):e0013137. doi: 10.1371/journal.pntd.0013137 (PMC12140206; doi:10.1371/journal.pntd.0013137)
Supplement: S4 File — (DOCX) [file pntd.0013137.s004.docx]

L.tropica

>Variant_1

ATGTCCGTGGACAGCAGCAGCACGCACCGGCGCCGCTGCGTCGCCGCGCGCCTGGTGCGCCTCGCGGCTGCCGGCGCCGCCGTCACCGTTGCTGTCGGCACCGCGGCCGCGTGGGCACACGCCGGCGCGCTGCAGCACCGCTGCATCCACGACGCGATGCAGGCACGCGTGCGGCAGTCGGTGGCGCGCCACCACACGGCCCCCGGCGCGGTGTCCGCGGTGGGCCTGCCGTACGTTACTCTCGACGCCGCGCACACCGCGGCCGCCGCCGATCCCAGGCCGGGCAGCGCGCCCACAGTCGTGCGCGCGGCGAACTGGGGCGCCCTGCGCGTCGCCGTCTCCACCGAGGACCTCACCGACCCCGCCTACCACTGCGCTCGCGTCGGACAGCGTGTCCACAACCACGCCGACCGCTTCGCCACCTGCACCGCCGAGGACATCCTCACCGACGAGAAGCGCGACATCCTCAGGAAATACCTCATCCCGCAGGCGCTGCAGCTGCACACGGAGCGGCTGAAGGCGCGGCAGGTGCAGGGCAAGTGGAAGGTGACGGGCATGGTCGACGAGATCTGTGGCGACTTCAAGGTGCCGCAGGCGCACATCACCGAGGGCTTCAGCAACACCGACTTCGTGATGTACGTCGCCTCCGTGCCGAGTGAGGAGGGTGTGCTGGCGTGGGCCACGACCTGCCAGGTGTTCTCTGACGGCCATCCAGCCGTGGGCGTCATCAACATCCCCGCGGCGAACATTGCGTCGCGGTACGACCAGGCCGTCACGCGTGTCGTCACGCACGAGATGGCGCACGCGCTCGGCTTCAGCGGCACATTCTTCGAAGACGCCCGCATCGTGGCGAGCATTTCGAACGTGCGGCAGAAGGGCTTCGATGTTCCCGTGATCAACAGCAGCACGGCGGTGGCGAAGGCGCGCGAGCAGTACGGCTGCGGCACCTTGGAGTATCTGGAGATAGAGGACCAGGGCAGTGCGGGCTCCGCCGGGTCGCACATCAAGATGCGCAACGCGCAGGACGAGCTCATGGCGGCAGCCAGTGGTGCCGGGTACTACACCGCCCTGACCATGGCCGTCTTCCAGGACCTCGGCTTCTACCAGGCGGACTTCAGCAAGGCGGAGGTGATGCCGTGGGGCCGGGACGCCGGCTGCGCCTTCCTCAGCGAGAAGTGCATGGAGGACGGCACCACGAAGTGGCCGGCGATGTTCTGCAACAGCGATGACGCCATCCGCTGCCCCACCAGTCGTCTCAGCCTGGGAACGTGTGGTGTTACCCATCAACCGGGCCTTCCGCCGTACTGGCAGTACTTCACGGACCCGTCCCTCGCCGGCGTCTCCGCCTTCATGGACTACTGCCCTGTCGTGGTGCCCTACGATGATGGCAGCTGTGCGCAGCGTGCCTCTGAGGCACATGCTTCGTTGCTGCCCTTCAACGTCTTCTCCGACGCGGCGCGCTGCATCGATGGCGCCTTCAGGCCGAAGGCGACTCACGGCCTAATCAAGTCGTACGCCGGCCTGTGCGCCAACGTGCGGTGCGACACGGCCACGCGCACGTACAGCGTGCAGGTGCACGGCGGCAAGGGCTACGCCAACTGCACGCCGGGCCTCAGAGTTGAGCTGAGCACCGTGAGCAACGCCTTCGAGGAGGGCGGCTACATCACGTGCCCGCCGTACGTGGAGGTGTGCCAGGGCAACGTGCAGGCTGCCAAGGACGGCGGCAACGCCGCGGCTGGTCGCCGTGGTCCGCGCGCCGCGGCGACGGCGCTGTTGGTGGCCGCGCTGCTGGCCGTGGCGCTCTAG

>Variant_2

ATGTCCGTGGACAGCAGCAGCACGCACCGGCGCCGCTGCGTCGCCGCGCGCCTGGTGCGCCTCGCGGCTGCCGGCGCCGCCGTCACCGTTGTTGTCGGCACCGCGGCCGCGTGGGCACACGCCGGCGCGCTGCAGCACCGCTGCATCCACGACGCGATGCAGGCACGCGTGCGGCAGTCGGTGGCGCGCCACCACACGGCCCCCGGCGCGGTGTCCGCGGTGGGCCTGCCGTACGTTACTCTCGACGCCGCGCACACCGCGGCCGCCGCCGATCCCAGGCCGGGCAGCGCGCCCACAGTCGTGCGCGCGGCGAACTGGGGCGCCCTGCGCGTCGCCGTCTCCACCGAGGACCTCACCGACCCCGCCTACCACTGCGCTCGCGTCGGACAGCGTGTCCACAACCACGCCGACCGCTTCGCCACCTGCACCGCCGAGGACATCCTCACCGACGAGAAGCGCGACATCCTCAGGAAATACCTCATCCCGCAGGCGCTGCAGCTGCACACGGAGCGGCTGAAGGCGCGGCAGGTGCAGGGCAAGTGGAAGGTGACGGGCATGGTCGACGAGATCTGTGGCGACTTCAAGGTGCCGCAGGCGCACATCACCGAGGGCTTCAGCAACACCGACTTCGTGATGTACGTCGCCTCCGTGCCGAGTGAGGAGGGTGTGCTGGCGTGGGCCACCACCTGCCAGGTGTTCTCTGACGGCCATCCAGCCGTGGGCGTCATCAACATCCCCGCGGCGAACATTGCGTCGCGGTACGACCAGGCCGTCACGCGTGTCGTCACGCACGAGATGGCGCACGCGCTCGGCTTCAGCGGCACATTCTTCGAAGACGCCCGCATCGTGGCGAGCATTTCGAACGTGCGGCAGAAGGGCTTCGATGTTCCCGTGATCAACAGCAGCACGGCGGTGGCGAAGGCGCGCGAGCAGTACGGCTGCGGCACCTTGGAGTATCTGGAGATAGAGGACCAGGGCAGTGCGGGCTCCGCCGGGTCGCACATCAAGATGCGCAACGCGCAGGACGAGCTCATGGCGGCAGCCAGTGGTGCCGGGTACTACACCGCCCTGACCATGGCCGTCTTCCAGGACCTCGGCTTCTACCAGGCGGACTTCAGCAAGGCGGAGGTGATGCCGTGGGGCCGGGACGCCGGCTGCGCCTTCCTCAGCGAGAAGTGCATGGAGGACGGCACCACGAAGTGGCCGGCGATGTTCTGCAACAGCGATGACGCCATCCGCTGCCCCACCAGTCGTCTCAGCCTGGGAACGTGTGGTGTTACCCATCAACCGGGCCTTCCGCCGTACTGGCAGTACTTCACGGACCCGTCCCTCGCCGGCGTCTCCGCCTTCATGGACTACTGCCCTGTCGTGGTGCCCTACGATGATGGCAGCTGTGCGCAGCGTGCCTCTGAGGCACATGCTTCGTTGCTGCCCTTCAACGTCTTCTCCGACGCGGCGCGCTGCATCGATGGCGCCTTCAGGCCGAAGACGACTCACGGCCTAATCAAGTCGTACGCCGGCCTGTGCGCCAACGTGCGGTGCGACACGGCCACGCGCACGTACAGCGTGCAGGTGCACGGCGGCAAGGGCTACGCCAACTGCACGCCGGGCCTCAGAGTTGAGCTGAGCACCGTGAGCAACGCCTTCGAGGAGGGCGGCTACATCACGTGCCCGCCGTACGTGGAGGTGTGCCAGGGCAACGTGCAGGCTGCCAAGGACGGCGGCAACGCCGCGGCTGGTCGCCGTGGTCCGCGCGCCGCGGCGACGGCGCTGTTGGTGGCCGCGCTGCTGGCCGTGGCGCTCTAG

>Variant_3

ATGTCCGTGGACAGCAGCAGCACGCACCGGCGCCGCTGCGTCGCCGCGCGCCTGGTGCGCCTCGCGGCTGCCGGCGCCGCCGTCACCGTTGCTGTCGGCACCGCGGCCGCGTGGGCACACGCCGGCGCGCTGCAGCACCGCTGCATCCACGACGCGATGCAGGCACGCGTGCGGCAGTCGGTGGCGCGCCACCACACGGCCCCCGGCGCGGTGTCCGCGGTGGGGCTGCCGTACGTTACTCTCGACGCCGCGCACACCGCGGCCGCCGCCGATCCCAGGCCGGGCAGCGCGCCCACAGTCGTGCGCGCGGCGAACTGGGGCGCCCTGCGCATCGCCGTCTCCACCGAGGACCTCACCGACCCCGCCTACCACTGCGCTCGCGTCGGACAGCGTGTCCACAACCACGCCGACCGCTTCGCCACCTGCACCGCCGAGGACATCCTCACCGACGAGAAGCGCGACATCCTCAGGAAATACCTCATCCCGCAGGCGCTGCTGCTGCACACGGAGCGGCTGAAGGCGCGGCAGGTGCAGGGCAAGTGGAAGGTGACGGGCATGGTCGACGACATCTGTGGCGACTTCAAGGTGCCGCAGGCGCACATCACCGAGGGCTTCAGCAACACCGACTTCGTGATGTACGTCGCCTCCGTGCCGAGTGAGGAGGGTGTGCTGGCGTGGGCCACCACCTGCCAGGTGTTCTCTGACGACCATCCAGCCGTGGGCGTCATCATCATCCCCGCGGCGAACATTGCGTCGCGGTACGACCAGGACGTCACGCGTGTCGTCACGCACGAGATGGCGCACGCGCTCGGCTTCAGCGGCACATTCTTCGAAGACGACCGCATCGTGGCGAGCATTTCGAACGTGCGGCAGAAGGACTTCGATGTTCCCGTGATCAACAGCAGCACGGCGGTGGCGAAGGCGCGCGAGCAGTACGGCTGCGGCACCTTGGAGTATCTGGAGATAGAGGACCAGGGCGGTGCGGGCTCCGCCGGGTCGCACATCAAGATGCGCAACGCGCAGGACGAGCTCATGGCGGCAGCCAGTGGTGCCGGGTACTACACCGCCCTGACCATGGCCGTCTTCCAGGACCTCGGCTTCTACCAGGCGGACTTCAGCAAGGCGGAGGTGATGCCGTGGGGCCGGGACGCCGGCTGCGCCTTCCTCAGCGAGAAGTGCATGGAGGACGGCACCACGAAGTGGCCGGCGATGTTCTGCAACAGCGATGACGCCATCCGCTGCCCCACCAGTCGTCTCAGCCTGGGAACGTGTGGTGTTACCCATCAACCGGGCCTTCCGCCGTACTGGCAGTACTTCACGGACCCGTCCCTCGCCGGCGTCTCCGCCTTCATGGACTACTGCCCTGTCGTGGAGCCCTACGATGATGGCAGCTGTGCGCAGCGTGCCTCTGAGGCACATGCTTCGTTGCTGCCCTTCAACGTCTTCTCCGACGCGGCGCGCTGCATCGATGGCGCCTTCAGGCCGAAGGCGACTCACGGCCTAATCAAGTCGTACGCCGGCCTGTGCGCCAACGTGCGGTGCGACACGGCCACGCGCACGTACAGCGTGCAGGTGCACGGCGGCAAGGGCTACGCCAACTGCACGCCGGGCCTCAGAGTTGAGCTGAGCACCGTGAGCAACGCCTTCGAGGAGGGCGGCTACATCACGTGCCCGCCGTACGTGGAGGTGTGCCAGGGCAACGTGCAGGCTGCCAAGGACGGCGGCAACGCCGCGGCTGGTCGCCGTGGTCCGCGCGCCGCGGCGACGGCGCTGTTGGTGGCCGCGCTGCTGGCCGTGGCGCTCTAG

>Variant_4

ATGTCCGTGGACAGCAGCAGCACGCACCGGCGCCGCTGCGTCGCCGCGCGCCTGGTGCGCCTCGCGGCTGCCGGCGCCGCCGTCACCGTTGCTGTCGGCACCGCGGCCGCGTGGGCACACGCCGGCGCGCTGCAGCACCGCTGCATCCACGACGCGATGCAGGCACGCGTGCGGCAGTCGGTGGCGCGCCACCACACGGCCCCCGGCGCGGTGTCGGCGGTGGTGCTGCCGTACGTTACTCTCGACGCCGCGCACACCGCGGCCGCCGCCGATCCCAGGCCGGGCAGCGCGCCCACAGTCGTGCGCGCGGCGAACTGGGGCGCCCTGCTCATCGCCGTCTCCACCGAGGACCTCACCGACCCCGCCTACCACTGCGCTCGCGTCGGACAGCGTGTCCACAACCACGACGACCGCTTCGCCACCTGCACCGCCGAGGACATCCTCACCGACGAGAAGCGCGACATCCTCAGGAAATACCTCATCCCGCAGGCGCTGCTGCTGCACACGGAGCGGCTGAAGGCGCGGCAGGTGCAGGGCAAGTGGAAGGTGACGGGCATGGTCGACGACATCTGTGGCGACTTCAAGGTGCCGCAGGCGCACATCACCGAGGGCTTCAGCAACACCGACTTCGTGATGTACGTCGCCTCCGTGCCGAGTGAGGAGGGTGTGCTGGCGTGGGCCACCACCTGCCAGGTGTTCTCTGACGACCATCCAGCCGTGGGCGTCATCAACATCCCCGCGGCGAACATTGCGTCGCGGTACGACCAGGACGTCACGCGTGTCGTCACGCACGAGATGGCGCACGCGCTCGGCTTCAGCGGCACATTCTTCGAAGACGCCCGCATCGTGGCGAGCATTTCGAACGTGCGGCAGAAGGGCTTCGATGTTCCCGTGATCAACAGCAGCACGGCGGTGGCGAAGGCGCGCGAGCAGTACGGCTGCGGCACCTTGGAGTATCTGGAGATAGAGGACCAGGGCGGTGCGGGCTCCGCCGGGTCGCACATCAAGATGCGCAACGCGCAGGACGAGCTCATGGCGGCAGCCAGTGGTGCCGGGTACTACACCGCCCTGACCATGGCCGTCTTCCAGGACCTCGGCTTCTACCAGGCGGACTTCAGCAAGGCGGAGGTGATGCCGTGGGGCCGGGACGCCGGCTGCGCCTTCCTCAGCGAGAAGTGCATGGAGGACGGCACCACGAAGTGGCCGGCGATGTTCTGCAACAGCGATGACGCCATCCGCTGCCCCACCAGTCGTCTCAGCCTGGGAACGTGTGGTGTTACCCATCAACCGGGCCTTCCGCCGTACTGGCAGTACTTCACGGACCCGTCCCTCGCCGGCGTCTCCGCCTTCATGGACTACTGCCCTGTCGTGGTGCCCTACGATGATGGCAGCTGTGCGCAGCGTGCCTCTGAGGCACATGCTTCGTTGCTGCCCTTCAACGTCTTCTCCGACGCGGCGCGCTGCATCGATGGCGCCTTCAGGCCGAAGACGACTCACGGCCTAATCAAGTCGTACGCCGGCCTGTGCGCCAACGTGCGGTGCGACACGGCCACGCGCACGTACAGCGTGCAGGTGCACGGCGGCAAGGGCTACGCCAACTGCACGCCGGGCCTCAGAGTTGAGCTGAGCACCGTGAGCAACGCCTTCGAGGAGGGCGGCTACATCACGTGCCCGCCGTACGTGGAGGTGTGCCAGGGCAACGTGCAGGCTGCCAAGGACGGCGGCAACGCCGCGGCTGGTCGCCGTGGTCCGCGCGCCGCGGCGACGGCGCTGTTGGTGGCCGCGCTGCTGGCCGTGGCGCTCTAG

>Variant_5

ATGTCCGTGGACAGCAGCAGCACGCACCGGCGCCGCTGCGTCGCCGCGCGCCTGGTGCGCCTCGCGGCTGCCGGCGCCGCCGTCACCGTTGCTGTCGGCACCGCGGCCGCGTGGGCACACGCCGGCGCGCTGCAGCACCGCTGCATCCACGACGCGATGCAGGCACGCGTGCGGCAGTCGGTGGCGCGCCACCACACGGCCCCCGGCGCGGTGTCCGCGGTGGGGCTGCCGTACGTTATTCTCGACGCCGCGCACACCGCGGCCGCCGCCGATCCCAGGCCGGGCAGCGCGCCCACAGTCGTGCGCGCGGCGAACTGGGGCGCCCTGCGCATCGCCGTCTCCACCGAGGACCTCACCGACCCCGCCTACCACTGCGCTCGCGTCGGACAGCGTGTCCACAACCACGCCGACCGCTTCGCCACCTGCACCGCCGAGGACATCCTCACCGACGAGAAGCGCGACATCCTCAGGAAATACCTCATCCCGCAGGCGCTGCAGCTGCACACGGAGCGGCTGAAGGCGCGGCAGGTGCAGGGCAAGTGGAAGGTGACGGGCATGGTCGACGAGATCTGTGGCGACTTCAAGGTGCCGCAGGCGCACATCACCGAGGGCTTCAGCAACACCGACTTCGTGATGTACGTCGCCTCCGTGCCGAGTGAGGAGGGTGTGCTGGCGTGGGCCACCACCTGCCAGGTGTTCTCTGACGGCCATCCAGCCGTGGGCGTCATCAACATCCCCGCGGCGAACATTGCGTCGCGGTACGACCAGGACGTCACGCGTGTCGTCACGCACGAGATGGCGCACGCGCTCGGCTTCAGCGGCACATTCTTCGAAGACGCCCGCATCGTGGCGAGCATTTCGAACGTGCGGCAGAAGGACTTCGATGTTCCCGTGATCAACAGCAGCACGGCGGTGGCGAAGGCGCGCGAGCAGTACGGCTGCGGCACCTTGGAGTATCTGGAGATAGAGGACCAGGGCGGTGCGGGCTCCGCCGGGTCGCACATCATGATGCGCAACGCGCAGGACGAGCTCATGGCGGCAGCCAGTGGTGCCGGGTACTACACCGCCCTGACCATGGCCGTCTTCCAGGACCTCGGCTTCTACCAGGCGGACTTCAGCAAGGCGGAGGTGATGCCGTGGGGCCGGGACGCCGGCTGCGCCTTCCTCAGCGAGAAGTGCATGGAGGAGGGCATCACGAAGTGGCCGGCGATGTTCTGCAACAGCGATGACGCCATCCGCTGCCCCACCAGTCGTCTCAGCCTGGGAACGTGTGGTGTTACCCATCAACCGGGCCTTCCGCCGTACTGGCAGTACTTCACGGACCCGTCCCTCGCCGGCGTCTCCGCCTTCATGGACTACTGCCCTGTCGTGGAGCCCTACGATGATGGCAGCTGTGCGCAGCGTGCCTCTGAGGCACATGCTTCGTTGCTGCCCTTCAACGTCTTCTCCGACGCGGCGCGCTGCATCGATGGCGCCTTCAGGCCGAAGACGACTCACGGCCTAATCAAGTCGTACGCCGGCCTGTGCGCCAACGTGCGGTGCGACACGGCCACGCGCACGTACAGCGTGCAGGTGCACGGCGGCAAGGGCTACGCCAACTGCACGCCGGGCCTCAGAGTTGAGCTGAGCACCGTGAGCAACGCCTTCGAGGAGGGCGGCTACATCACGTGCCCGCCGTACGTGGAGGTGTGCCAGGGCAACGTGCAGGCTGCCAAGGACGGCGGCAACGCCGCGGCTGGTCGCCGTGGTCCGCGCGCCGCGGCGACGGCGCTGTTGGTGGCCGCGCTGCTGGCCGTGGCGCTCTAG

>Variant_6

ATGTCCGTGGACAGCAGCAGCACGCACCGGCGCCGCTGCGTCGCCGCGCGCCTGGTGCGCCTCGCGGCTGCCGGCGCCGCCGTCACCGTTGCTGTCGGCACCGCGGCCGCGTGGGCACACGCCGGCGCGCTGCAGCACCGCTGCATCCACGACGCGATGCAGGCACGCGTGCGGCAGTCGGTGGCGCGCCACCACACGGCCCCCGGCGCGGTGTCCGCGGTGGGCCTGCCGTACGTTACTCTCGACGCCGCGCACACCGCGGCCGCCGCCGATCCCAGGCCGGGCAGCGCGCCCACAGTCGTGCGCGCGGCGAACTGGGGCGCCCTGCGCATCGCCGTCTCCACCGAGGACCTCACCGACCCCGCCTACCACTGCGCTCGCGTCGGACAGCGTGTCCACAACCACGCCGACCGCTTCGCCACCTGCACCGCCGAGGACATCCTCACCGACGAGAAGCGCGACATCCTCAGGAAATACCTCATCCCGCAGGCGCTGCAGCTGCACACGGAGCGGCTGAAGGCGCGGCAGGTGCAGGGCAAGTGGAAGGTGACGGGCATGGTCGACGAGATCTGTGGCGACTTCAAGGTGCCGCAGGCGCACATCACCGAGGGCTTCAGCAACACCGACTTCGTGATGTACGTCGCCTCCGTGCCGAGTGAGGAGGGTGTGCTGGCGTGGGCCACGACCTGCCAGGTGTTCTCTGACGGCCATCCAGCCGTGGGCGTCATCATCATCCCCGCGGCGAACATTGCGTCGCGGTACGACCAGGACGTCACGCGTGTCGTCACGCACGAGATGGCGCACGCGCTCGGCTTCAGCGGCACATTCTTCGAAGACGCCCGCATCGTGGCGAGCATTTCGAACGTGCGGCAGAAGGACTTCGATGTTCCCGTGATCAACAGCAGCACGGCGGTGGCGAAGGCGCGCGAGCAGTACGGCTGCGGCACCTTGGAGTATCTGGAGATAGAGGACCAGGGCGGTGCGGGCTCCGCCGGGTCGCACATCATGATGCGCAACGCGCAGGACGAGCTCATGGCGGCAGCCAGTGGTGCCGGGTACTACACCGCCCTGACCATGGCCGTCTTCCAGGACCTCGGCTTCTACCAGGCGGACTTCAGCAAGGCGGAGGTGATGCCGTGGGGCCGGGACGCCGGCTGCGCCTTCCTCAGCGAGAAGTGCATGGAGCAGGGCATCACGAAGTGGCCGGCGATGTTCTGCAACAGCGATGACGCCATCCGCTGCCCCACCAGTCGTCTCAGCCTGGGAACGTGTGGTGTTACCCATCAACCGGGCCTTCCGCCGTACTGGCAGTACTTCACGGACCCGTCCCTCGCCGGCGTCTCCGCCTTCATGGACTACTGCCCTGTCGTGGAGCCCTACGATGATGGCAGCTGTGCGCAGCGTGCCTCTGAGGCACATGCTTCGTTGCTGCCCTTCAACGTCTTCTCCGACGCGGCGCGCTGCATCGATGGCGCCTTCAGGCCGAAGACGACTCACGGCCTAATCAAGTCGTACGCCGGCCTGTGCGCCAACGTGCGGTGCGACACGGCCACGCGCACGTACAGCGTGCAGGTGCACGGCGGCAAGGGCTACGCCAACTGCACGCCGGGCCTCAGAGTTGAGCTGAGCACCGTGAGCAACGCCTTCGAGGAGGGCGGCTACATCACGTGCCCGCCGTACGTGGAGGTGTGCCAGGGCAACGTGCAGGCTGCCAAGGACGGCGGCAACGCCGCGGCTGGTCGCCGTGGTCCGCGCGCCGCGGCGACGGCGCTGTTGGTGGCCGCGCTGCTGGCCGTGGCGCTCTAG

>Variant_7

ATGTCCGTGGACAGCAGCAGCACGCACCGGCGCCGCTGCGTCGCCGCGCGGCTGGTGCGCCTCGCGGCTGCCGGCGCCGCCGTCACCGTTGCTGTCGGCACCGCGGCCGCGTGGGCACACGCCGGCGCGCTGCAGCACCGCTGCATCCACGACACGATGCAGGCACGCGTGCGGCAGTCGGTGGCGCGCCACCACACGGCCCCCGGCGCGGTGTCGGCGGTGGTGCTGCCGTACGTTATTCTCGACGCCGCGCACACCGCGGCCGCCGACGATCCCAGGCCGGGCAGCGCGCCCACAGTCGTGCGCGCGGCGAACTGGGGCGCCCTGCTCATCGCCGTCTCCACCGAGGACCTCACCGACCCCGCCTACCACTGCGCTCGCGTCGGACAGCGTGTCCACAACCACGACGACCGCTTCGCCACCTGCACCGCCGAGGACATCCTCACCGACGAGAAGCGCGACATCCTCAGGAAATACCTCATCCCGCAGGCGCTGCAGCTGCACACGGAGCGGCTGAAGGCGCGGCAGGTGCAGGGCAAGTGGAAGGTGACGGGCATGGTCGACGAGATCTGTGGCGACTTCAAGGTGCCGCAGGCGCACATCACCGAGGGCTTCAGCAACACCGACTTCGTGATGTACGTCGCCTCCGTGCCGAGTGAGGAGGGTGTGCTGGCGTGGGCCACGACCTGCCAGGTGTTCTCTGACGGCCATCCAGCCGTGGGCGTCATCAACATCCCCGCGGCGAACATTGCGTCGCGGTACGACCAGGCCGTCACGCGTGTCGTCACGCACGAGATGGCGCACGCGCTCGGCTTCAGCGGCACATTCTTCGAAGACGCCCGCATCGTGGCGAGCATTTCGAACGTGCGGCAGAAGGGCTTCGATGTTCCCGTGATCAACAGCAGCACGGCGGTGGCGAAGGCGCGCGAGCAGTACGGCTGCGGCACCTTGGAGTATCTGGAGATAGAGGACCAGGGCGGTGCGGGCTCCGCCGGGTCGCACATCAAGATGCGCAACGCGCAGGACGAGCTCATGGCGGCAGCCAGTGGTGCCGGGTACTACACCGCCCTGACCATGGCCGTCTTCCAGGACCTCGGCTTCTACCAGGCGGACTTCAGCAAGGCGGAGGTGATGCCGTGGGGCCGGGACGCCGGCTGCGCCTTCCTCAGCGAGAAGTGCATGGAGGACGGCACCACGAAGTGGCCGGCGATGTTCTGCAACAGCGATGACGCCATCCGCTGCCCCACCAGTCGTCTCAGCCTGGGAACGTGTGGTGTTACCCATCAACCGGGCCTTCCGCCGTACTGGCAGTACTTCACGGACCCGTCCCTCGCCGGCGTCTCCGCCTTCATGGACTACTGCCCTGTCGTGGAGCCCTACGATGATGGCAGCTGTGCGCAGCGTGCCTCTGAGGCACATGCTTCGTTGCTGCCCTTCAACGTCTTCTCCGACGCGGCGCGCTGCATCGATGGCGCCTTCAGGCCGAAGGCGACTCACGGCCTAATCAAGTCGTACGCCGGCCTGTGCGCCAACGTGCGGTGCGACACGGCCACGCGCACGTACAGCGTGCAGGTGCACGGCGGCAAGGGCTACGCCAACTGCACGCCGGGCCTCAGAGTTGAGCTGAGCACCGTGAGCAACGCCTTCGAGGAGGGCGGCTACATCACGTGCCCGCCGTACGTGGAGGTGTGCCAGGGCAACGTGCAGGCTGCCAAGGACGGCGGCAACGCCGCGGCTGGTCGCCGTGGTCCGCGCGCCGCGGCGACGGCGCTGTTGGTGGCCGCGCTGCTGGCCGTGGCGCTCTAG

>Variant_8

ATGTCCGTGGACAGCAGCAGCACGCACCGGCGCCGCTGCGTCGCCGCGCGGCTGGTGCGCCTCGCGGCTGCCGGCGCCGCCGTCGCCGTTGCTGTCGGCACCGCGGCCGCGTGGGCACACGCCGGCGCGCTGCAGCACCGCTGCATCCACGACGCGATGCAGGCACGCGTGCGGCAGTCGGTGGCGCGCCACCACACGGCCCCCGGCGCGGTGTCCGCGGTGGGGCTGCCGTACGTTACTCTCGACGCCGCGCACACCGCGGCCGCCGACGATCCCAGGCCGGGCAGCGCGCCCACAGTCGTGCGCGCGGCGAACTGGGGCGCCCTGCTCATCGCCGTCTCCACCGAGGACCTCACCGACCCCGCCTACCACTGCGCTCGCGTCGGACAGCGTGTCCACAACCACGACGACCGCTTCGCCACCTGCACCGCCGAGGACATCCTCACCGACGAGAAGCGCGACATCCTCAGGAAATACCTCATCCCGCAGGCGCTGCAGCTGCACACGGAGCGGCTGAAGGCGCGGCAGGTGCAGGGCAAGTGGAAGGTGACGGGCATGGTCGACGAGATCTGTGGCGACTTCAAGGTGCCGCAGGCGCACATCACCGAGGGCTTCAGCAACACCGACTTCGTGATGTACGTCGCCTCCGTGCCGAGTGAGGAGGGTGTGCTGGCGTGGGCCACGACCTGCCAGGTGTTCTCTGACGGCCATCCAGCCGTGGGCGTCATCAACATCCCCGCGGCGAACATTGCGTCGCGGTACGACCAGGCCGTCACGCGTGTCGTCACGCACGAGATGGCGCACGCGCTCGGCTTCAGCGGCACATTCTTCGAAGACGCCCGCATCGTGGCGAGCATTTCGAACGTGCGGCAGAAGGGCTTCGATGTTCCCGTGATCAACAGCAGCACGGCGGTGGCGAAGGCGCGCGAGCAGTACGGCTGCGGCACCTTGGAGTATCTGGAGATAGAGGACCAGGGCGGTGCGGGCTCCGCCGGGTCGCACATCAAGATGCGCAACGCGCAGGACGAGCTCATGGCGGCAGCCAGTGGTGCCGGGTACTACACCGCCCTGACCATGGCCGTCTTCCAGGACCTCGGCTTCTACCAGGCGGACTTCAGCAAGGCGGAGGTGATGCCGTGGGGCCGGGACGCCGGCTGCGCCTTCCTCAGCGAGAAGTGCATGGAGGACGGCACCACGAAGTGGCCGGCGATGTTCTGCAACAGCGATGACGCCATCCGCTGCCCCACCAGTCGTCTCAGCCTGGGAACGTGTGGTGTTACCCATCAACCGGGCCTTCCGCCGTACTGGCAGTACTTCACGGACCCGTCCCTCGCCGGCGTCTCCGCCTTCATGGACTACTGCCCTGTCGTGGAGCCCTACGATGATGGCAGCTGTGCGCAGCGTGCCTCTGAGGCACATGCTTCGTTGCTGCCCTTCAACGTCTTCTCCGACGCGGCGCGCTGCATCGATGGCGCCTTCAGGCCGAAGACGACTCACGGCCTAATCAAGTCGTACGCCGGCCTGTGCGCCAACGTGCGGTGCGACACGGCCACGCGCACGTACAGCGTGCAGGTGCACGGCGGCAAGGGCTACGCCAACTGCACGCCGGGCCTCAGAGTTGAGCTGAGCACCGTGAGCAACGCCTTCGAGGAGGGCGGCTACATCACGTGCCCGCCGTACGTGGAGGTGTGCCAGGGCAACGTGCAGGCTGCCAAGGACGGCGGCAACGCCGCGGCTGGTCGCCGTGGTCCGCGCGCCGCGGCGACGGCGCTGTTGGTGGCCGCGCTGCTGGCCGTGGCGCTCTAG

>Variant_9

ATGTCCGTGGACAGCAGCAGCACGCACCGGCGCCGCTGCGTCGCCGCGCGGCTGGTGCGCCTCGCGGCTGCCGGCGCCGCCGTCACCGTTGCTGTCGGCACCGCGGCCGCGTGGGCACACGCCGGCGCGCTGCAGCACCGCTGCATCCACGACACGATGCAGGCACGCGTGCGGCAGTCGGTGGCGCGCCACCACACGGCCCCCGGCGCGGTGTCGGCGGTGGTGCTGCCGTACGTTACTCTCGACGCCGCGCACACCGCGGCCGCCGACGATCCCAGGCCGGGCAGCGCGCCCACAGTCGTGCGCGCGGCGAACTGGGGCGCCCTGCGCGTCGCCGTCTCCACCGAGGACCTCACCGACCCCGCCTACCACTGCGCTCGCGTCGGACAGCGTGTCCACAACCACGACGACCGCTTCGCCACCTGCACCGCCGAGGACATCCTCACCGACGAGAAGCGCGACATCCTCAGGAAATACCTCATCCCGCAGGCGCTGCAGCTGCACACGGAGCGGCTGAAGGCGCGGCAGGTGCAGGGCAAGTGGAAGGTGACGGGCATGGTCGACGAGATCTGTGGCGACTTCAAGGTGCCGCAGGCGCACATCACCGAGGGCTTCAGCAACACCGACTTCGTGATGTACGTCGCCTCCGTGCCGAGTGAGGAGGGTGTGCTGGCGTGGGCCACCACCTGCCAGGTGTTCTCTGACGGCCATCCAGCCGTGGGCGTCATCATCATCCCCGCGGCGAACATTGCGTCGCGGTACGACCAGGACGTCACGCGTGTCGTCACGCACGAGATGGCGCACGCGCTCGGCTTCAGCGGCACATTCTTCGAAGACGCCCGCATCGTGGCGAGCATTTCGAACGTGCGGCAGAAGGACTTCGATGTTCCCGTGATCAACAGCAGCACGGCGGTGGCGAAGGCGCGCGAGCAGTACGGCTGCGGCACCTTGGAGTATCTGGAGATAGAGGACCAGGGCGGTGCGGGCTCCGCCGGGTCGCACATCATGATGCGCAACGCGCAGGACGAGCTCATGGCGGCAGCCAGTGGTGCCGGGTACTACACCGCCCTGACCATGGCCGTCTTCCAGGACCTCGGCTTCTACCAGGCGGACTTCAGCAAGGCGGAGGTGATGCCGTGGGGCCGGGACGCCGGCTGCGCCTTCCTCAGCGAGAAGTGCATGGAGCAGGGCACCACGAAGTGGCCGGCGATGTTCTGCAACAGCGATGACGCCATCCGCTGCCCCACCAGTCGTCTCAGCCTGGGAACGTGTGGTGTTACCCATCAACCGGGCCTTCCGCCGTACTGGCAGTACTTCACGGACCCGTCCCTCGCCGGCGTCTCCGCCTTCATGGACTACTGCCCTGTCGTGGTGCCCTACGATGATGGCAGCTGTGCGCAGCGTGCCTCTGAGGCACATGCTTCGTTGCTGCCCTTCAACGTCTTCTCCGACGCGGCGCGCTGCATCGATGGCGCCTTCAGGCCGAAGACGACTCACGGCCTAATCAAGTCGTACGCCGGCCTGTGCGCCAACGTGCGGTGCGACACGGCCACGCGCACGTACAGCGTGCAGGTGCACGGCGGCAAGGGCTACGCCAACTGCACGCCGGGCCTCAGAGTTGAGCTGAGCACCGTGAGCAACGCCTTCGAGGAGGGCGGCTACATCACGTGCCCGCCGTACGTGGAGGTGTGCCAGGGCAACGTGCAGGCTGCCAAGGACGGCGGCAACGCCGCGGCTGGTCGCCGTGGTCCGCGCGCCGCGGCGACGGCGCTGTTGGTGGCCGCGCTGCTGGCCGTGGCGCTCTAG

>Variant_10

ATGTCCGTGGACAGCAGCAGCACGCACCGGCGCCGCTGCGTCGCCGCGCGCCTGGTGCGCCTCGCGGCTGCCGGCGCCGCCGTCACCGTTGTTGTCGGCACCGCGGCCGCGTGGGCACACGCCGGCGCGCTGCAGCACCGCTGCATCCACGACGCGATGCAGGCACGCGTGCGGCAGTCGGTGGCGCGCCACCACACGGCCCCCGGCGCGGTGTCCGCGGTGGGGCTGCCGTACGTTACTCTCGACGCCGCGCACACCGCGGCCGCCGCCGATCCCAGGCCGGGCAGCGCGCCCACAGTCGTGCGCGCGGCGAACTGGGGCGCCCTGCGCATCGCCGTCTCCACCGAGGACCTCACCGACCCCGCCTACCACTGCGCTCGCGTCGGACAGCGTGTCCACAACCACGCCGACCGCTTCGCCACCTGCACCGCCGAGGACATCCTCACCGACGAGAAGCGCGACATCCTCAGGAAATACCTCATCCCGCAGGCGCTGCAGCTGCACACGGAGCGGCTGAAGGCGCGGCAGGTGCAGGGCAAGTGGAAGGTGACGGGCATGGTCGACGAGATCTGTGGCGACTTCAAGGTGCCGCAGGCGCACATCACCGAGGGCTTCAGCAACACCGACTTCGTGATGTACGTCGCCTCCGTGCCGAGTGAGGAGGGTGTGCTGGCGTGGGCCACGACCTGCCAGGTGTTCTCTGACGGCCATCCAGCCGTGGGCGTCATCAACATCCCCGCGGCGAACATTGCGTCGCGGTACGACCAGGCCGTCACGCGTGTCGTCACGCACGAGATGGCGCACGCGCTCGGCTTCAGCGGCACATTCTTCGAAGACGCCCGCATCGTGGCGAGCATTTCGAACGTGCGGCAGAAGGGCTTCGATGTTCCCGTGATCAACAGCAGCACGGCGGTGGCGAAGGCGCGCGAGCAGTACGGCTGCGGCACCTTGGAGTATCTGGAGATAGAGGACCAGGGCAGTGCGGGCTCCGCCGGGTCGCACATCAAGATGCGCAACGCGCAGGACGAGCTCATGGCGGCAGCCAGTGGTGCCGGGTACTACACCGCCCTGACCATGGCCGTCTTCCAGGACCTCGGCTTCTACCAGGCGGACTTCAGCAAGGCGGAGGTGATGCCGTGGGGCCGGGACGCCGGCTGCGCCTTCCTCAGCGAGAAGTGCATGGAGGACGGCACCACGAAGTGGCCGGCGATGTTCTGCAACAGCGATGACGCCATCCGCTGCCCCACCAGTCGTCTCAGCCTGGGAACGTGTGGTGTTACCCATCAACCGGGCCTTCCGCCGTACTGGCAGTACTTCACGGACCCGTCCCTCGCCGGCGTCTCCGCCTTCATGGACTACTGCCCTGTCGTGGTGCCCTACGATGATGGCAGCTGTGCGCAGCGTGCCTCTGAGGCACATGCTTCGTTGCTGCCCTTCAACGTCTTCTCCGACGCGGCGCGCTGCATCGATGGCGCCTTCAGGCCGAAGGCGACTCACGGCCTAATCAAGTCGTACGCCGGCCTGTGCGCCAACGTGCGGTGCGACACGGCCACGCGCACGTACAGCGTGCAGGTGCACGGCGGCAAGGGCTACGCCAACTGCACGCCGGGCCTCAGAGTTGAGCTGAGCACCGTGAGCAACGCCTTCGAGGAGGGCGGCTACATCACGTGCCCGCCGTACGTGGAGGTGTGCCAGGGCAACGTGCAGGCTGCCAAGGACGGCGGCAACGCCGCGGCTGGTCGCCGTGGTCCGCGCGCCGCGGCGACGGCGCTGTTGGTGGCCGCGCTGCTGGCCGTGGCGCTCTAG

>Variant_11

ATGTCCGTGGACAGCAGCAGCACGCACCGGCGCCGCTGCGTCGCCGCGCGCCTGGTGCGCCTCGCGGCTGCCGGCGCCGCCGTCACCGTTGCTGTCGGCACCGCGGCCGCGTGGGCACACGCCGGCGCGCTGCAGCACCGCTGCATCCACGACGCGATGCAGGCACGCGTGCGGCAGTCGGTGGCGCGCCACCACACGGCCCCCGGCGCGGTGTCCGCGGTGGGGCTGCCGTACGTTACTCTCGACGCCGCGCACACCGCGGCCGCCGCCGATCCCAGGCCGGGCAGCGCGCCCACAGTCGTGCGCGCGGCGAACTGGGGCGCCCTGCGCATCGCCGTCTCCACCGAGGACCTCACCGACCCCGCCTACCACTGCGCTCGCGTCGGACAGCGTGTCCACAACCACGCCGACCGCTTCGCCACCTGCACCGCCGAGGACATCCTCACCGACGAGAAGCGCGACATCCTCAGGAAATACCTCATCCCGCAGGCGCTGCAGCTGCACACGGAGCGGCTGAAGGCGCGGCAGGTGCAGGGCAAGTGGAAGGTGACGGGCATGGTCGACGAGATCTGTGGCGACTTCAAGGTGCCGCAGGCGCACATCACCGAGGGCTTCAGCAACACCGACTTCGTGATGTACGTCGCCTCCGTGCCGAGTGAGGAGGGTGTGCTGGCGTGGGCCACGACCTGCCAGGTGTTCTCTGACGGCCATCCAGCCGTGGGCGTCATCAACATCCCCGCGGCGAACATTGCGTCGCGGTACGACCAGGCCGTCACGCGTGTCGTCACGCACGAGATGGCGCACGCGCTCGGCTTCAGCGGCACATTCTTCGAAGACGCCCGCATCGTGGCGAGCATTTCGAACGTGCGGCAGAAGGGCTTCGATGTTCCCGTGATCAACAGCAGCACGGCGGTGGCGAAGGCGCGCGAGCAGTACGGCTGCGGCACCTTGGAGTATCTGGAGATAGAGGACCAGGGCAGTGCGGGCTCCGCCGGGTCGCACATCAAGATGCGCAACGCGCAGGACGAGCTCATGGCGGCAGCCAGTGGTGCCGGGTACTACACCGCCCTGACCATGGCCGTCTTCCAGGACCTCGGCTTCTACCAGGCGGACTTCAGCAAGGCGGAGGTGATGCCGTGGGGCCGGGACGCCGGCTGCGCCTTCCTCAGCGAGAAGTGCATGGAGGACGGCACCACGAAGTGGCCGGCGATGTTCTGCAACAGCGATGACGCCATCCGCTGCCCCACCAGTCGTCTCAGCCTGGGAACGTGTGGTGTTACCCATCAACCGGGCCTTCCGCCGTACTGGCAGTACTTCACGGACCCGTCCCTCGCCGGCGTCTCCGCCTTCATGGACTACTGCCCTGTCGTGGTGCCCTACGATGATGGCAGCTGTGCGCAGCGTGCCTCTGAGGCACATGCTTCGTTGCTGCCCTTCAACGTCTTCTCCGACGCGGCGCGCTGCATCGATGGCGCCTTCAGGCCGAAGGCGACTCACGGCCTAATCAAGTCGTACGCCGGCCTGTGCGCCAACGTGCGGTGCGACACGGCCACGCGCACGTACAGCGTGCAGGTGCACGGCGGCAAGGGCTACGCCAACTGCACGCCGGGCCTCAGAGTTGAGCTGAGCACCGTGAGCAACGCCTTCGAGGAGGGCGGCTACATCACGTGCCCGCCGTACGTGGAGGTGTGCCAGGGCAACGTGCAGGCTGCCAAGGACGGCGGCAACGCCGCGGCTGGTCGCCGTGGTCCGCGCGCCGCGGCGACGGCGCTGTTGGTGGCCGCGCTGCTGGCCGTGGCGCTCTAG

>Variant_12

ATGTCCGTGGACAGCAGCAGCACGCACCGGCGCCGCTGCGTCGCCGCGCGGCTGGTGCGCCTCGCGGCTGCCGGCGCCGCCGTCGCCGTTGCTGTCGGCACCGCGGCCGCGTGGGCACACGCCGGCGCGCTGCAGCACCGCTGCATCCACGACGCGATGCAGGCACGCGTGCGGCAGTCGGTGGCGCGCCACCACACGGCCCCCGGCGCGGTGTCCGCGGTGGGGCTGCCGTACGTTACTCTCGACGCCGCGCACACCGCGGCCGCCGCCGATCCCAGGCCGGGCAGCGCGCCCACAGTCGTGCGCGCGGCGAACTGGGGCGCCCTGCGCATCGCCGTCTCCACCGAGGACCTCACCGACCCCGCCTACCACTGCGCTCGCGTCGGACAGCGTGTCCACAACCACGCCGACCGCTTCGCCACCTGCACCGCCGAGGACATCCTCACCGACGAGAAGCGCGACATCCTCAGGAAATACCTCATCCCGCAGGCGCTGCAGCTGCACACGGAGCGGCTGAAGGCGCGGCAGGTGCAGGGCAAGTGGAAGGTGACGGGCATGGTCGACGAGATCTGTGGCGACTTCAAGGTGCCGCAGGCGCACATCACCGAGGGCTTCAGCAACACCGACTTCGTGATGTACGTCGCCTCCGTGCCGAGTGAGGAGGGTGTGCTGGCGTGGGCCACGACCTGCCAGGTGTTCTCTGACGGCCATCCAGCCGTGGGCGTCATCAACATCCCCGCGGCGAACATTGCGTCGCGGTACGACCAGGACGTCACGCGTGTCGTCACGCACGAGATGGCGCACGCGCTCGGCTTCAGCGGCACATTCTTCGAAGACGCCCGCATCGTGGCGAGCATTTCGAACGTGCGGCAGAAGGACTTCGATGTTCCCGTGATCAACAGCAGCACGGCGGTGGCGAAGGCGCGCGAGCAGTACGGCTGCGGCACCTTGGAGTATCTGGAGATAGAGGACCAGGGCGGTGCGGGCTCCGCCGGGTCGCACATCAAGATGCGCAACGCGCAGGACGAGCTCATGGCGGCAGCCAGTGGTGCCGGGTACTACACCGCCCTGACCATGGCCGTCTTCCAGGACCTCGGCTTCTACCAGGCGGACTTCAGCAAGGCGGAGGTGATGCCGTGGGGCCGGGACGCCGGCTGCGCCTTCCTCAGCGAGAAGTGCATGGAGCAGGGCATCACGAAGTGGCCGGCGATGTTCTGCAACAGCGATGACGCCATCCGCTGCCCCACCAGTCGTCTCAGCCTGGGAACGTGTGGTGTTACCCATCAACCGGGCCTTCCGCCGTACTGGCAGTACTTCACGGACCCGTCCCTCGCCGGCGTCTCCGCCTTCATGGACTACTGCCCTGTCGTGGAGCCCTACGATGATGGCAGCTGTGCGCAGCGTGCCTCTGAGGCACATGCTTCGTTGCTGCCCTTCAACGTCTTCTCCGACGCGGCGCGCTGCATCGATGGCGCCTTCAGGCCGAAGACGACTCACGGCCTAATCAAGTCGTACGCCGGCCTGTGCGCCAACGTGCGGTGCGACACGGCCACGCGCACGTACAGCGTGCAGGTGCACGGCGGCAAGGGCTACGCCAACTGCACGCCGGGCCTCAGAGTTGAGCTGAGCACCGTGAGCAACGCCTTCGAGGAGGGCGGCTACATCACGTGCCCGCCGTACGTGGAGGTGTGCCAGGGCAACGTGCAGGCTGCCAAGGACGGCGGCAACGCCGCGGCTGGTCGCCGTGGTCCGCGCGCCGCGGCGACGGCGCTGTTGGTGGCCGCGCTGCTGGCCGTGGCGCTCTAG

>Variant_13

ATGTCCGTGGACAGCAGCAGCACGCACCGGCGCCGCTGCGTCGCCGCGCGCCTGGTGCGCCTCGCGGCTGCCGGCGCCGCCGTCACCGTTGTTGTCGGCACCGCGGCCGCGTGGGCACACGCCGGCGCGCTGCAGCACCGCTGCATCCACGACGCGATGCAGGCACGCGTGCGGCAGTCGGTGGCGCGCCACCACACGGCCCCCGGCGCGGTGTCCGCGGTGGTGCTGCCGTACGTTACTCTCGACGCCGCGCACACCGCGGCCGCCGCCGATCCCAGGCCGGGCAGCGCGCCCACAGTCGTGCGCGCGGCGAACTGGGGCGCCCTGCTCATCGCCGTCTCCACCGAGGACCTCACCGACCCCGCCTACCACTGCGCTCGCGTCGGACAGCGTGTCCACAACCACGCCGACCGCTTCGCCACCTGCACCGCCGAGGACATCCTCACCGACGAGAAGCGCGACATCCTCAGGAAATACCTCATCCCGCAGGCGCTGCAGCTGCACACGGAGCGGCTGAAGGCGCGGCAGGTGCAGGGCAAGTGGAAGGTGACGGGCATGGTCGACGAGATCTGTGGCGACTTCAAGGTGCCGCAGGCGCACATCACCGAGGGCTTCAGCAACACCGACTTCGTGATGTACGTCGCCTCCGTGCCGAGTGAGGAGGGTGTGCTGGCGTGGGCCACGACCTGCCAGGTGTTCTCTGACGGCCATCCAGCCGTGGGCGTCATCAACATCCCCGCGGCGAACATTGCGTCGCGGTACGACCAGGACGTCACGCGTGTCGTCACGCACGAGATGGCGCACGCGCTCGGCTTCAGCGGCACATTCTTCGAAGACGCCCGCATCGTGGCGAGCATTTCGAACGTGCGGCAGAAGGACTTCGATGTTCCCGTGATCAACAGCAGCACGGCGGTGGCGAAGGCGCGCGAGCAGTACGGCTGCGGCACCTTGGAGTATCTGGAGATAGAGGACCAGGGCGGTGCGGGCTCCGCCGGGTCGCACATCATGATGCGCAACGCGCAGGACGAGCTCATGGCGGCAGCCAGTGGTGCCGGGTACTACACCGCCCTGACCATGGCCGTCTTCCAGGACCTCGGCTTCTACCAGGCGGACTTCAGCAAGGCGGAGGTGATGCCGTGGGGCCGGGACGCCGGCTGCGCCTTCCTCAGCGAGAAGTGCATGGAGCAGGGCATCACGAAGTGGCCGGCGATGTTCTGCAACAGCGATGACGCCATCCGCTGCCCCACCAGTCGTCTCAGCCTGGGAACGTGTGGTGTTACCCATCAACCGGGCCTTCCGCCGTACTGGCAGTACTTCACGGACCCGTCCCTCGCCGGCGTCTCCGCCTTCATGGACTACTGCCCTGTCGTGGTGCCCTACGATGATGGCAGCTGTGCGCAGCGTGCCTCTGAGGCACATGCTTCGTTGCTGCCCTTCAACGTCTTCTCCGACGCGGCGCGCTGCATCGATGGCGCCTTCAGGCCGAAGGCGACTCACGGCCTAATCAAGTCGTACGCCGGCCTGTGCGCCAACGTGCGGTGCGACACGGCCACGCGCACGTACAGCGTGCAGGTGCACGGCGGCAAGGGCTACGCCAACTGCACGCCGGGCCTCAGAGTTGAGCTGAGCACCGTGAGCAACGCCTTCGAGGAGGGCGGCTACATCACGTGCCCGCCGTACGTGGAGGTGTGCCAGGGCAACGTGCAGGCTGCCAAGGACGGCGGCAACGCCGCGGCTGGTCGCCGTGGTCCGCGCGCCGCGGCGACGGCGCTGTTGGTGGCCGCGCTGCTGGCCGTGGCGCTCTAG

>Variant_14

ATGTCCGTGGACAGCAGCAGCACGCACCGGCGCCGCTGCGTCGCCGCGCGGCTGGTGCGCCTCGCGGCTGCCGGCGCCGCCGTCACCGTTGCTGTCGGCACCGCGGCCGCGTGGGCACACGCCGGCGCGCTGCAGCACCGCTGCATCCACGACACGATGCAGGCACGCGTGCGGCAGTCGGTGGCGCGCCACCACACGGCCCCCGGCGCGGTGTCCGCGGTGGGGCTGCCGTACGTTACTCTCGACGCCGCGCACACCGCGGCCGCCGCCGATCCCAGGCCGGGCAGCGCGCCCACAGTCGTGCGCGCGGCGAACTGGGGCGCCCTGCGCATCGCCGTCTCCACCGAGGACCTCACCGACCCCGCCTACCACTGCGCTCGCGTCGGACAGCGTGTCCACAACCACGCCGACCGCTTCGCCACCTGCACCGCCGAGGACATCCTCACCGACGAGAAGCGCGACATCCTCAGGAAATACCTCATCCCGCAGGCGCTGCAGCTGCACACGGAGCGGCTGAAGGCGCGGCAGGTGCAGGGCAAGTGGAAGGTGACGGGCATGGTCGACGAGATCTGTGGCGACTTCAAGGTGCCGCAGGCGCACATCACCGAGGGCTTCAGCAACACCGACTTCGTGATGTACGTCGCCTCCGTGCCGAGTGAGGAGGGTGTGCTGGCGTGGGCCACCACCTGCCAGGTGTTCTCTGACGGCCATCCAGCCGTGGGCGTCATCAACATCCCCGCGGCGAACATTGCGTCGCGGTACGACCAGGACGTCACGCGTGTCGTCACGCACGAGATGGCGCACGCGCTCGGCTTCAGCGGCACATTCTTCGAAGACGCCCGCATCGTGGCGAGCATTTCGAACGTGCGGCAGAAGGACTTCGATGTTCCCGTGATCAACAGCAGCACGGCGGTGGCGAAGGCGCGCGAGCAGTACGGCTGCGGCACCTTGGAGTATCTGGAGATAGAGGACCAGGGCGGTGCGGGCTCCGCCGGGTCGCACATCATGATGCGCAACGCGCAGGACGAGCTCATGGCGGCAGCCAGTGGTGCCGGGTACTACACCGCCCTGACCATGGCCGTCTTCCAGGACCTCGGCTTCTACCAGGCGGACTTCAGCAAGGCGGAGGTGATGCCGTGGGGCCGGGACGCCGGCTGCGCCTTCCTCAGCGAGAAGTGCATGGAGCAGGGCACCACGAAGTGGCCGGCGATGTTCTGCAACAGCGATGACGCCATCCGCTGCCCCACCAGTCGTCTCAGCCTGGGAACGTGTGGTGTTACCCATCAACCGGGCCTTCCGCCGTACTGGCAGTACTTCACGGACCCGTCCCTCGCCGGCGTCTCCGCCTTCATGGACTACTGCCCTGTCGTGGAGCCCTACGATGATGGCAGCTGTGCGCAGCGTGCCTCTGAGGCACATGCTTCGTTGCTGCCCTTCAACGTCTTCTCCGACGCGGCGCGCTGCATCGATGGCGCCTTCAGGCCGAAGACGACTCACGGCCTAATCAAGTCGTACGCCGGCCTGTGCGCCAACGTGCGGTGCGACACGGCCACGCGCACGTACAGCGTGCAGGTGCACGGCGGCAAGGGCTACGCCAACTGCACGCCGGGCCTCAGAGTTGAGCTGAGCACCGTGAGCAACGCCTTCGAGGAGGGCGGCTACATCACGTGCCCGCCGTACGTGGAGGTGTGCCAGGGCAACGTGCAGGCTGCCAAGGACGGCGGCAACGCCGCGGCTGGTCGCCGTGGTCCGCGCGCCGCGGCGACGGCGCTGTTGGTGGCCGCGCTGCTGGCCGTGGCGCTCTAG

>Variant_15

ATGTCCGTGGACAGCAGCAGCACGCACCGGCGCCGCTGCGTCGCCGCGCGGCTGGTGCGCCTCGCGGCTGCCGGCGCCGCCGTCACCGTTGCTGTCGGCACCGCGGCCGCGTGGGCACACGCCGGCGCGCTGCAGCACCGCTGCATCCACGACGCGATGCAGGCACGCGTGCGGCAGTCGGTGGCGCGCCACCACACGGCCCCCGGCGCGGTGTCCGCGGTGGGGCTGCCGTACGTTACTCTCGACGCCGCGCACACCGCGGCCGCCGCCGATCCCAGGCCGGGCAGCGCGCCCACAGTCGTGCGCGCGGCGAACTGGGGCGCCCTGCGCATCGCCGTCTCCACCGAGGACCTCACCGACCCCGCCTACCACTGCGCTCGCGTCGGACAGCGTGTCCACAACCACGCCGACCGCTTCGCCACCTGCACCGCCGAGGACATCCTCACCGACGAGAAGCGCGACATCCTCAGGAAATACCTCATCCCGCAGGCGCTGCAGCTGCACACGGAGCGGCTGAAGGCGCGGCAGGTGCAGGGCAAGTGGAAGGTGACGGGCATGGTCGACGAGATCTGTGGCGACTTCAAGGTGCCGCAGGCGCACATCACCGAGGGCTTCAGCAACACCGACTTCGTGATGTACGTCGCCTCCGTGCCGAGTGAGGAGGGTGTGCTGGCGTGGGCCACGACCTGCCAGGTGTTCTCTGACGGCCATCCAGCCGTGGGCGTCATCAACATCCCCGCGGCGAACATTGCGTCGCGGTACGACCAGGACGTCACGCGTGTCGTCACGCACGAGATGGCGCACGCGCTCGGCTTCAGCGGCACATTCTTCGAAGACGCCCGCATCGTGGCGAGCATTTCGAACGTGCGGCAGAAGGACTTCGATGTTCCCGTGATCAACAGCAGCACGGCGGTGGCGAAGGCGCGCGAGCAGTACGGCTGCGGCACCTTGGAGTATCTGGAGATAGAGGACCAGGGCGGTGCGGGCTCCGCCGGGTCGCACATCATGATGCGCAACGCGCAGGACGAGCTCATGGCGGCAGCCAGTGGTGCCGGGTACTACACCGCCCTGACCATGGCCGTCTTCCAGGACCTCGGCTTCTACCAGGCGGACTTCAGCAAGGCGGAGGTGATGCCGTGGGGCCGGGACGCCGGCTGCGCCTTCCTCAGCGAGAAGTGCATGGAGGACGGCACCACGAAGTGGCCGGCGATGTTCTGCAACAGCGATGACGCCATCCGCTGCCCCACCAGTCGTCTCAGCCTGGGAACGTGTGGTGTTACCCATCAACCGGGCCTTCCGCCGTACTGGCAGTACTTCACGGACCCGTCCCTCGCCGGCGTCTCCGCCTTCATGGACTACTGCCCTGTCGTGGAGCCCTACGATGATGGCAGCTGTGCGCAGCGTGCCTCTGAGGCACATGCTTCGTTGCTGCCCTTCAACGTCTTCTCCGACGCGGCGCGCTGCATCGATGGCGCCTTCAGGCCGAAGACGACTCACGGCCTAATCAAGTCGTACGCCGGCCTGTGCGCCAACGTGCGGTGCGACACGGCCACGCGCACGTACAGCGTGCAGGTGCACGGCGGCAAGGGCTACGCCAACTGCACGCCGGGCCTCAGAGTTGAGCTGAGCACCGTGAGCAACGCCTTCGAGGAGGGCGGCTACATCACGTGCCCGCCGTACGTGGAGGTGTGCCAGGGCAACGTGCAGGCTGCCAAGGACGGCGGCAACGCCGCGGCTGGTCGCCGTGGTCCGCGCGCCGCGGCGACGGCGCTGTTGGTGGCCGCGCTGCTGGCCGTGGCGCTCTAG

>Variant_16

ATGTCCGTGGACAGCAGCAGCACGCACCGGCGCCGCTGCGTCGCCGCGCGGCTGGTGCGCCTCGCGGCTGCCGGCGCCGCCGTCACCGTTGTTGTCGGCACCGCGGCCGCGTGGGCACACGCCGGCGCGCTGCAGCACCGCTGCATCCACGACGCGATGCAGGCACGCGTGCGGCAGTCGGTGGCGCGCCACCACACGGCCCCCGGCGCGGTGTCGGCGGTGGTGCTGCCGTACGTTACTCTCGACGCCGCGCACACCGCGGCCGCCGACGATCCCAGGCCGGGCAGCGCGCCCACAGTCGTGCGCGCGGCGAACTGGGGCGCCCTGCGCATCGCCGTCTCCACCGAGGACCTCACCGACCCCGCCTACCACTGCGCTCGCGTCGGACAGCGTGTCCACAACCACGCCGACCGCTTCGCCACCTGCACCGCCGAGGACATCCTCACCGACGAGAAGCGCGACATCCTCAGGAAATACCTCATCCCGCAGGCGCTGCTGCTGCACACGGAGCGGCTGAAGGCGCGGCAGGTGCAGGGCAAGTGGAAGGTGACGGGCATGGTCGACGACATCTGTGGCGACTTCAAGGTGCCGCAGGCGCACATCACCGAGGGCTTCAGCAACACCGACTTCGTGATGTACGTCGCCTCCGTGCCGAGTGAGGAGGGTGTGCTGGCGTGGGCCACCACCTGCCAGGTGTTCTCTGACGGCCATCCAGCCGTGGGCGTCATCAACATCCCCGCGGCGAACATTGCGTCGCGGTACGACCAGGACGTCACGCGTGTCGTCACGCACGAGATGGCGCACGCGCTCGGCTTCAGCGGCACATTCTTCGAAGACGACCGCATCGTGGCGAGCATTTCGAACGTGCGGCAGAAGGACTTCGATGTTCCCGTGATCAACAGCAGCACGGCGGTGGCGAAGGCGCGCGAGCAGTACGGCTGCGGCACCTTGGAGTATCTGGAGATAGAGGACCAGGGCGGTGCGGGCTCCGCCGGGTCGCACATCATGATGCGCAACGCGCAGGACGAGCTCATGGCGGCAGCCAGTGGTGCCGGGTACTACACCGCCCTGACCATGGCCGTCTTCCAGGACCTCGGCTTCTACCAGGCGGACTTCAGCAAGGCGGAGGTGATGCCGTGGGGCCGGGACGCCGGCTGCGCCTTCCTCAGCGAGAAGTGCATGGAGGACGGCACCACGAAGTGGCCGGCGATGTTCTGCAACAGCGATGACGCCATCCGCTGCCCCACCAGTCGTCTCAGCCTGGGAACGTGTGGTGTTACCCATCAACCGGGCCTTCCGCCGTACTGGCAGTACTTCACGGACCCGTCCCTCGCCGGCGTCTCCGCCTTCATGGACTACTGCCCTGTCGTGGAGCCCTACGATGATGGCAGCTGTGCGCAGCGTGCCTCTGAGGCACATGCTTCGTTGCTGCCCTTCAACGTCTTCTCCGACGCGGCGCGCTGCATCGATGGCGCCTTCAGGCCGAAGGCGACTCACGGCCTAATCAAGTCGTACGCCGGCCTGTGCGCCAACGTGCGGTGCGACACGGCCACGCGCACGTACAGCGTGCAGGTGCACGGCGGCAAGGGCTACGCCAACTGCACGCCGGGCCTCAGAGTTGAGCTGAGCACCGTGAGCAACGCCTTCGAGGAGGGCGGCTACATCACGTGCCCGCCGTACGTGGAGGTGTGCCAGGGCAACGTGCAGGCTGCCAAGGACGGCGGCAACGCCGCGGCTGGTCGCCGTGGTCCGCGCGCCGCGGCGACGGCGCTGTTGGTGGCCGCGCTGCTGGCCGTGGCGCTCTAG

>Variant_17

ATGTCCGTGGACAGCAGCAGCACGCACCGGCGCCGCTGCGTCGCCGCGCGGCTGGTGCGCCTCGCGGCTGCCGGCGCCGCCGTCACCGTTGTTGTCGGCACCGCGGCCGCGTGGGCACACGCCGGCGCGCTGCAGCACCGCTGCATCCACGACGCGATGCAGGCACGCGTGCGGCAGTCGGTGGCGCGCCACCACACGGCCCCCGGCGCGGTGTCCGCGGTGGTGCTGCCGTACGTTACTCTCGACGCCGCGCACACCGCGGCCGCCGACGATCCCAGGCCGGGCAGCGCGCCCACAGTCGTGCGCGCGGCGAACTGGGGCGCCCTGCGCATCGCCGTCTCCACCGAGGACCTCACCGACCCCGCCTACCACTGCGCTCGCGTCGGACAGCGTGTCCACAACCACGCCGACCGCTTCGCCACCTGCACCGCCGAGGACATCCTCACCGACGAGAAGCGCGACATCCTCAGGAAATACCTCATCCCGCAGGCGCTGCAGCTGCACACGGAGCGGCTGAAGGCGCGGCAGGTGCAGGGCAAGTGGAAGGTGACGGGCATGGTCGACGAGATCTGTGGCGACTTCAAGGTGCCGCAGGCGCACATCACCGAGGGCTTCAGCAACACCGACTTCGTGATGTACGTCGCCTCCGTGCCGAGTGAGGAGGGTGTGCTGGCGTGGGCCACCACCTGCCAGGTGTTCTCTGACGGCCATCCAGCCGTGGGCGTCATCAACATCCCCGCGGCGAACATTGCGTCGCGGTACGACCAGGACGTCACGCGTGTCGTCACGCACGAGATGGCGCACGCGCTCGGCTTCAGCGGCACATTCTTCGAAGACGCCCGCATCGTGGCGAGCATTTCGAACGTGCGGCAGAAGGGCTTCGATGTTCCCGTGATCAACAGCAGCACGGCGGTGGCGAAGGCGCGCGAGCAGTACGGCTGCGGCACCTTGGAGTATCTGGAGATAGAGGACCAGGGCGGTGCGGGCTCCGCCGGGTCGCACATCAAGATGCGCAACGCGCAGGACGAGCTCATGGCGGCAGCCAGTGGTGCCGGGTACTACACCGCCCTGACCATGGCCGTCTTCCAGGACCTCGGCTTCTACCAGGCGGACTTCAGCAAGGCGGAGGTGATGCCGTGGGGCCGGGACGCCGGCTGCGCCTTCCTCAGCGAGAAGTGCATGGAGGACGGCACCACGAAGTGGCCGGCGATGTTCTGCAACAGCGATGACGCCATCCGCTGCCCCACCAGTCGTCTCAGCCTGGGAACGTGTGGTGTTACCCATCAACCGGGCCTTCCGCCGTACTGGCAGTACTTCACGGACCCGTCCCTCGCCGGCGTCTCCGCCTTCATGGACTACTGCCCTGTCGTGGAGCCCTACGATGATGGCAGCTGTGCGCAGCGTGCCTCTGAGGCACATGCTTCGTTGCTGCCCTTCAACGTCTTCTCCGACGCGGCGCGCTGCATCGATGGCGCCTTCAGGCCGAAGACGACTCACGGCCTAATCAAGTCGTACGCCGGCCTGTGCGCCAACGTGCGGTGCGACACGGCCACGCGCACGTACAGCGTGCAGGTGCACGGCGGCAAGGGCTACGCCAACTGCACGCCGGGCCTCAGAGTTGAGCTGAGCACCGTGAGCAACGCCTTCGAGGAGGGCGGCTACATCACGTGCCCGCCGTACGTGGAGGTGTGCCAGGGCAACGTGCAGGCTGCCAAGGACGGCGGCAACGCCGCGGCTGGTCGCCGTGGTCCGCGCGCCGCGGCGACGGCGCTGTTGGTGGCCGCGCTGCTGGCCGTGGCGCTCTAG

>Variant_18

ATGTCCGTGGACAGCAGCAGCACGCACCGGCGCCGCTGCGTCGCCGCGCGCCTGGTGCGCCTCGCGGCTGCCGGCGCCGCCGTCACCGTTGCTGTCGGCACCGCGGCCGCGTGGGCACACGCCGGCGCGCTGCAGCACCGCTGCATCCACGACGCGATGCAGGCACGCGTGCGGCAGTCGGTGGCGCGCCACCACACGGCCCCCGGCGCGGTGTCCGCGGTGGGCCTGCCGTACGTTACTCTCGACGCCGCGCACACCGCGGCCGCCGCCGATCCCAGGCCGGGCAGCGCGCCCACAGTCGTGCGCGCGGCGAACTGGGGCGCCCTGCGCATCGCCGTCTCCACCGAGGACCTCACCGACCCCGCCTACCACTGCGCTCGCGTCGGACAGCGTGTCCACAACCACGCCGACCGCTTCGCCACCTGCACCGCCGAGGACATCCTCACCGACGAGAAGCGCGACATCCTCAGGAAATACCTCATCCCGCAGGCGCTGCAGCTGCACACGGAGCGGCTGAAGGCGCGGCAGGTGCAGGGCAAGTGGAAGGTGACGGGCATGGTCGACGAGATCTGTGGCGACTTCAAGGTGCCGCAGGCGCACATCACCGAGGGCTTCAGCAACACCGACTTCGTGATGTACGTCGCCTCCGTGCCGAGTGAGGAGGGTGTGCTGGCGTGGGCCACGACCTGCCAGGTGTTCTCTGACGGCCATCCAGCCGTGGGCGTCATCAACATCCCCGCGGCGAACATTGCGTCGCGGTACGACCAGGCCGTCACGCGTGTCGTCACGCACGAGATGGCGCACGCGCTCGGCTTCAGCGGCACATTCTTCGAAGACGCCCGCATCGTGGCGAGCATTTCGAACGTGCGGCAGAAGGGCTTCGATGTTCCCGTGATCAACAGCAGCACGGCGGTGGCGAAGGCGCGCGAGCAGTACGGCTGCGGCACCTTGGAGTATCTGGAGATAGAGGACCAGGGCAGTGCGGGCTCCGCCGGGTCGCACATCAAGATGCGCAACGCGCAGGACGAGCTCATGGCGGCAGCCAGTGGTGCCGGGTACTACACCGCCCTGACCATGGCCGTCTTCCAGGACCTCGGCTTCTACCAGGCGGACTTCAGCAAGGCGGAGGTGATGCCGTGGGGCCGGGACGCCGGCTGCGCCTTCCTCAGCGAGAAGTGCATGGAGGACGGCACCACGAAGTGGCCGGCGATGTTCTGCAACAGCGATGACGCCATCCGCTGCCCCACCAGTCGTCTCAGCCTGGGAACGTGTGGTGTTACCCATCAACCGGGCCTTCCGCCGTACTGGCAGTACTTCACGGACCCGTCCCTCGCCGGCGTCTCCGCCTTCATGGACTACTGCCCTGTCGTGGTGCCCTACGATGATGGCAGCTGTGCGCAGCGTGCCTCTGAGGCACATGCTTCGTTGCTGCCCTTCAACGTCTTCTCCGACGCGGCGCGCTGCATCGATGGCGCCTTCAGGCCGAAGGCGACTCACGGCCTAATCAAGTCGTACGCCGGCCTGTGCGCCAACGTGCGGTGCGACACGGCCACGCGCACGTACAGCGTGCAGGTGCACGGCGGCAAGGGCTACGCCAACTGCACGCCGGGCCTCAGAGTTGAGCTGAGCACCGTGAGCAACGCCTTCGAGGAGGGCGGCTACATCACGTGCCCGCCGTACGTGGAGGTGTGCCAGGGCAACGTGCAGGCTGCCAAGGACGGCGGCAACGCCGCGGCTGGTCGCCGTGGTCCGCGCGCCGCGGCGACGGCGCTGTTGGTGGCCGCGCTGCTGGCCGTGGCGCTCTAG

>Variant_19

ATGTCCGTGGACAGCAGCAGCACGCACCGGCGCCGCTGCGTCGCCGCGCGGCTGGTGCGCCTCGCGGCTGCCGGCGCCGCCGTCACCGTTGTTGTCGGCACCGCGGCCGCGTGGGCACACGCCGGCGCGCTGCAGCACCGCTGCATCCACGACACGATGCAGGCACGCGTGCGGCAGTCGGTGGCGCGCCACCACACGGCCCCCGGCGCGGTGTCCGCGGTGGGGCTGCCGTACGTTATTCTCGACGCCGCGCACACCGCGGCCGCCGCCGATCCCAGGCCGGGCAGCGCGCCCACAGTCGTGCGCGCGGCGAACTGGGGCGCCCTGCTCATCGCCGTCTCCACCGAGGACCTCACCGACCCCGCCTACCACTGCGCTCGCGTCGGACAGCGTGTCCACAACCACGACGACCGCTTCGCCACCTGCACCGCCGAGGACATCCTCACCGACGAGAAGCGCGACATCCTCAGGAAATACCTCATCCCGCAGGCGCTGCTGCTGCACACGGAGCGGCTGAAGGCGCGGCAGGTGCAGGGCAAGTGGAAGGTGACGGGCATGGTCGACGACATCTGTGGCGACTTCAAGGTGCCGCAGGCGCACATCACCGAGGGCTTCAGCAACACCGACTTCGTGATGTACGTCGCCTCCGTGCCGAGTGAGGAGGGTGTGCTGGCGTGGGCCACCACCTGCCAGGTGTTCTCTGACGACCATCCAGCCGTGGGCGTCATCATCATCCCCGCGGCGAACATTGCGTCGCGGTACGACCAGGACGTCACGCGTGTCGTCACGCACGAGATGGCGCACGCGCTCGGCTTCAGCGGCACATTCTTCGAAGACGCCCGCATCGTGGCGAGCATTTCGAACGTGCGGCAGAAGGGCTTCGATGTTCCCGTGATCAACAGCAGCACGGCGGTGGCGAAGGCGCGCGAGCAGTACGGCTGCGGCACCTTGGAGTATCTGGAGATAGAGGACCAGGGCGGTGCGGGCTCCGCCGGGTCGCACATCAAGATGCGCAACGCGCAGGACGAGCTCATGGCGGCAGCCAGTGGTGCCGGGTACTACACCGCCCTGACCATGGCCGTCTTCCAGGACCTCGGCTTCTACCAGGCGGACTTCAGCAAGGCGGAGGTGATGCCGTGGGGCCGGGACGCCGGCTGCGCCTTCCTCAGCGAGAAGTGCATGGAGGACGGCACCACGAAGTGGCCGGCGATGTTCTGCAACAGCGATGACGCCATCCGCTGCCCCACCAGTCGTCTCAGCCTGGGAACGTGTGGTGTTACCCATCAACCGGGCCTTCCGCCGTACTGGCAGTACTTCACGGACCCGTCCCTCGCCGGCGTCTCCGCCTTCATGGACTACTGCCCTGTCGTGGAGCCCTACGATGATGGCAGCTGTGCGCAGCGTGCCTCTGAGGCACATGCTTCGTTGCTGCCCTTCAACGTCTTCTCCGACGCGGCGCGCTGCATCGATGGCGCCTTCAGGCCGAAGGCGACTCACGGCCTAATCAAGTCGTACGCCGGCCTGTGCGCCAACGTGCGGTGCGACACGGCCACGCGCACGTACAGCGTGCAGGTGCACGGCGGCAAGGGCTACGCCAACTGCACGCCGGGCCTCAGAGTTGAGCTGAGCACCGTGAGCAACGCCTTCGAGGAGGGCGGCTACATCACGTGCCCGCCGTACGTGGAGGTGTGCCAGGGCAACGTGCAGGCTGCCAAGGACGGCGGCAACGCCGCGGCTGGTCGCCGTGGTCCGCGCGCCGCGGCGACGGCGCTGTTGGTGGCCGCGCTGCTGGCCGTGGCGCTCTAG
